# Pyridoxine 5′-phosphate oxidase is a novel therapeutic target and regulated by the TGF-β signalling pathway in epithelial ovarian cancer

**DOI:** 10.1038/s41419-017-0050-3

**Published:** 2017-12-13

**Authors:** Lingyun Zhang, Daibing Zhou, Wencai Guan, Weimin Ren, Wenwen Sun, Jimin Shi, Qunbo Lin, Jinguo Zhang, Tiankui Qiao, Yulong Ye, Yun Wu, Yaning Zhang, Xulei Zuo, Kristin L Connor, Guoxiong Xu

**Affiliations:** 1 Center Laboratory, Jinshan Hospital, Fudan University, Shanghai, 201508 China; 2 Department of Oncology, Shanghai Medical College, Fudan University, Shanghai, 200032 China; 3 Department of Oncology, Jinshan Hospital, Fudan University, Shanghai, 201508 China; 4 Shanghai Jinshan District Center for Disease Control and Prevention, Shanghai, 201599 China; 5 Department of Obstetrics and Gynecology, Jinshan Hospital, Fudan University, Shanghai, 201508 China; 6 Department of Health Sciences, Carleton University, Ottawa, ON K1S 5B6 Canada

## Abstract

Pyridoxine 5′-phosphate oxidase (PNPO) is an enzyme that converts pyridoxine 5′-phosphate into pyridoxal 5′-phosphate (PLP), an active form of vitamin B6 implicated in several types of cancer. However, the role of PNPO and its regulatory mechanism in epithelial ovarian cancer (EOC) are unknown. In the present study, PNPO expression in human ovarian tumour tissue and its association with the clinicopathological features of patients with EOC were examined. Further, the biological function of PNPO in EOC cells and in xenograft was evaluated. We demonstrated for the first time that PNPO was overexpressed in human EOC. Knockdown of PNPO induced EOC cell apoptosis, arrested cell cycle at G2/M phase, decreased cell proliferation, migration and invasion. Xenografts of PNPO-shRNA-expressing cells into the nude mouse attenuated tumour growth. PNPO at mRNA and protein levels in EOC cells was decreased after transforming growth factor-β1 (TGF-β1) treatment. The inhibitory effect of TGF-β1 on PNPO expression was abolished in the presence of SB-431542, a TGF-β type I receptor kinase inhibitor. Moreover, we found that TGF-β1-mediated PNPO expression was at least in part through the upregulation of miR-143-3p. These data indicate a mechanism underlying PNPO regulation by the TGF-β signalling pathway. Furthermore, PLP administration reduced PNPO expression and decreased EOC cell proliferation, suggesting a feedback loop between PLP and PNPO. Thus, our findings reveal that PNPO can serve as a novel tissue biomarker of EOC and may be a potential target for therapeutic intervention.

## Introduction

Human ovarian cancer (OC) is the most deadly disease in women. Histologically, there are three main types of cancer: epithelial, sex cord-stromal and germ cell tumours^1–3^. Epithelial ovarian cancer (EOC), derived from the epithelial cells of the ovary or the fallopian tube^4^, accounts for more than 90% of total OC and occurs most commonly in postmenopausal women^5^. About 70% of EOCs are at an advanced stage because of an inability to detect the disease early due to an absence of symptoms and lack of an effective diagnostic marker^6,7^, making it the most lethal gynaecological malignancy. As such, there is a critical need to identify biomarkers for early detection of OC and possible targets for therapeutic intervention.

Vitamin B6 exist as six vitamers, including pyridoxine (PN), pyridoxamine (PM), pyridoxine 5′-phosphate (PNP), pyridoxamine 5′-phosphate (PMP), pyridoxal 5′-phosphate (PLP) and pyridoxal (PL)^8^. Dietary PN and PM serve as the main source of PNP and PMP. Oxidation of PNP and PMP produces PLP which can be further metabolized to PL through enzymatic hydrolysis^9,10^. PLP, an active form of vitamin B6, is an essential cofactor required by many enzymes for metabolic processes including metabolism of carbohydrates, fats and proteins^11–13^. Pyridoxine 5′-phosphate oxidase (PNP oxidase, PNPO), also known as PMP oxidase, is a key enzyme in vitamin B6 metabolism and converts PNP and PMP into PLP^14^. The *PNPO* gene is located on chromosome 17q21.32^15^ and the level of PNPO mRNA expression is relatively high in human liver, skeletal muscle and kidney, but low in lung and ovary^16^. PNPO has known to play a role in human epilepsy. PNPO deficiency, due to mutations in the *PNPO* gene, has been widely reported in neonatal/infantile epileptic encephalopathy^17,18^. Additionally, few reports indicate that PNPO has been implicated in breast and colorectal cancers^19–21^. However, it remains unknown whether PNPO plays a role in the development and progression of EOC.

Transforming growth factor-β (TGF-β) is an important cytokine involved in a variety of cellular processes and has been implicated in carcinogenesis^22^. TGF-β plays key roles in the regulation of many biological functions, including cell proliferation, migration, invasion and apoptosis and has dual actions in tumour suppression and tumour promotion under certain circumstances^23,24^. The TGF-β subfamily members (TGF-β1, TGF-β2 and TGF-β3) activate the downstream Smad transducer proteins, such as Smad2 and Smad3, by the heteromeric complexes of its type I (TβRI) and type II (TβRII) receptors^25,26^. Clinical studies showed that the dysregulation of TGF-β signalling may contribute to the development of OC and is associated with metastasis and survival^27,28^. However, whether TGF-β regulates PNPO expression in OC is largely unknown. Following our recent reports that human cystatin B, β-2-microglobulin and cytidine monophosphate kinase are ovarian tumour progression markers and are regulated by TGF-β1^29–31^, we speculate that PNPO may be another EOC biomarker and that it may also be regulated by TGF-β signalling.

In this study, we examine whether PNPO expression would be associated with clinicopathological features of patients with EOC and the change of its expression level would impact biological processes characteristic of cancer progression including cell proliferation, migration, invasion and apoptosis in vitro and tumour growth in vivo. We also examine the mechanism of how PNPO expression is regulated by the TGF-β signalling pathway.

## Results

### PNPO is overexpressed in human epithelial ovarian cancer

**Fig. 1:**
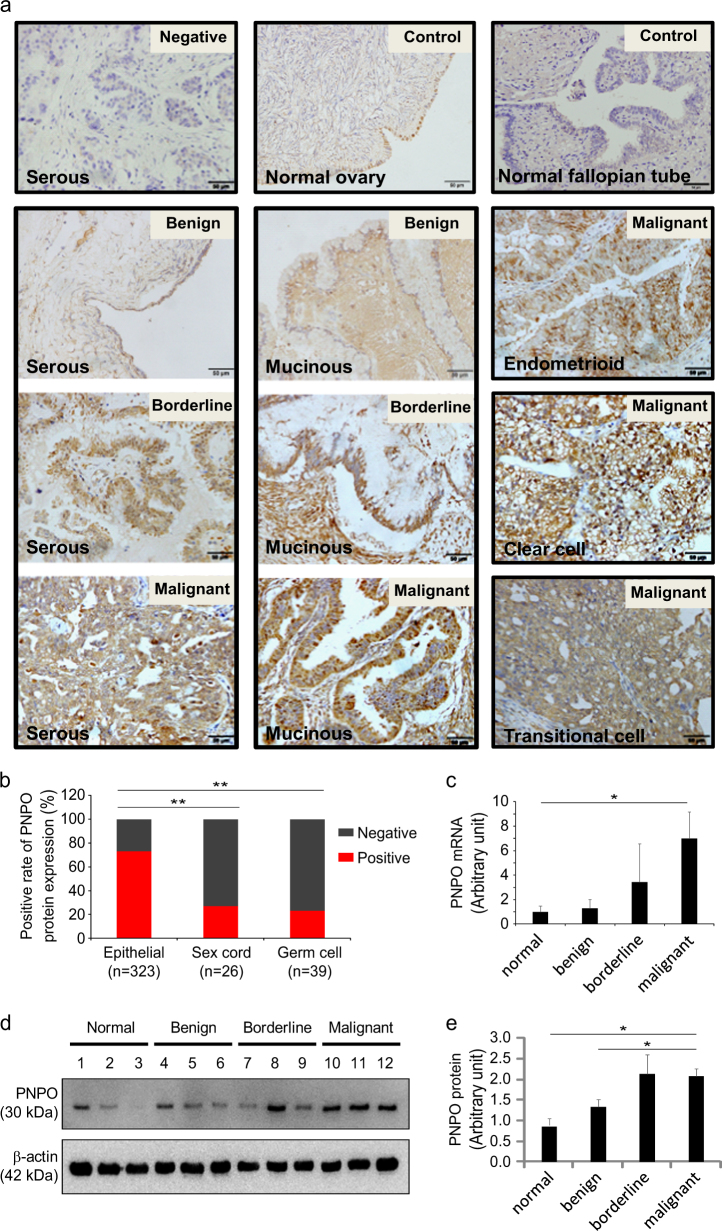
PNPO expression in human ovarian tissues **a** Immunohistochemistry staining of PNPO. Representative images of PNPO expression in the normal tissues of the fallopian tube and the ovary, serous and mucinous benign, borderline and malignant tumours, and endometrioid, clear cell and transitional cell malignant tumours are shown. A brown colour in an epithelial cell is considered as a positive staining. Original magnification, ×400. Scale bar, 50 µm. **b** Positive rate of PNPO protein expression in patients with ovarian tumours. Epithelial, surface epithelial-stromal tumours; Sex cord, sex cord-stromal tumours; Germ cell, germ cell tumours. **c** PNPO mRNA expression detected by qRT-PCR in the normal ovarian tissues, ovarian serous benign, borderline and malignant tumours (*n* = 3 each). **d** PNPO protein expression detected by western blot analysis in normal ovarian tissue (#1–3), ovarian serous benign tumours (#4–6), borderline tumours (#7–9) and malignant (#10–12) tumours. **e** Semi-quantitative analysis of the relative optical density of protein bands in **d**. Data are presented as mean ± SEM. *P < 0.05; **P < 0.01

Immunohistochemistry (IHC) showed that PNPO staining intensity was greater in human ovarian surface epithelial tumours (Fig. 1a and Supplementary Figure S1). The immunoreactive staining of PNPO was weak in normal tissues of the ovary and the fallopian tube and benign tumour, moderate in borderline tumour and strong in malignant tumours, including serous, mucinous, endometrioid, clear cell and transitional cell carcinomas. The positive staining was mainly distributed in the cytoplasm of the EOC cells. We also evaluated the association between PNPO protein expression and the clinical characteristics of patients with OC using tissue microarray (Fig. 1b and Supplementary Table S1). The positive rate of PNPO protein expression was slightly higher in patients at age > 45 *vs*. ⩽45 (*P* = 0.015). By analysis of histological types, we found that PNPO protein expression was significantly associated with surface epithelial malignant tumours compared with sex cord-stromal and germ cell tumours (*P* < 0.001, Fig. 1b). However, there was no association between subtypes of epithelial malignant tumours, including serous, mucinous and endometrioid adenocarcinomas, and clear and transitional cell carcinomas (Supplementary Table S1). PNPO protein expression was also affected by the grades (*P* = 0.024). Multiple comparisons showed that PNPO protein expression was not associated with the clinical stages of primary tumour site and FIGO or lymph node metastasis in patients with OC (*P* > 0.05). Next, we examined the expression of PNPO at mRNA and protein levels in epithelial serous tumours. The PNPO mRNA expression was higher in serous malignant tumour compared with the normal tissue of the ovary (*P* < 0.05, Fig. 1c). Overexpression of PNPO protein in ovarian serous malignant tumour was confirmed by western blot (Fig. 1d). Semi-quantitative analysis of the relative optical density of protein bands showed that the expression of PNPO protein was significantly high in malignant tumours than in the normal ovarian tissue and benign tumour (*P* < 0.05, Fig. 1e). Since the high-grade serous carcinoma may derive from the fallopian tube^32^, next we examined the expression levels of PNPO protein in the tissues of the normal fallopian tube and ovary as well as serous malignant tumours. Western blot analysis showed that the expression of PNPO protein was much higher in serous malignant tumours than the normal fallopian tube and ovarian tissues (*P* < 0.001, Supplementary Figure S2a and S2b). Furthermore, the expression of PNPO protein was higher in EOC cell lines OVCAR-3 and SK-OV-3, but not in other two adenocarcinoma cell lines CAOV-3 and A2780 and a clear cell line ES-2, compared with non-tumorous human ovarian surface epithelial cell line HOSEpiC (*P* < 0.05; Supplementary Figure S3a and S3b).

### Knockdown of PNPO inhibits ovarian cancer cell proliferation and arrests cell cycle

Since PNPO was overexpressed in EOC, a loss-of-function approach was used to evaluate the functional significance of PNPO in OVCAR-3 and SK-OV-3 cells. Quantitative real-time PCR (qRT-PCR) and western blot confirmed the efficacy of PNPO silencing at the mRNA and protein levels in cells transfected with PNPO-siRNA compared to cells transfected with negative control (NC) siRNA (Supplementary Figure S4a−S4d). Further, cell proliferation was significantly decreased after PNPO-siRNA transfection in OVCAR-3 cells for 72 h and in SK-OV-3 cells for 48 and 72 h (*P* < 0.05, Supplementary Figure S4e and S4f).

**Fig. 2:**
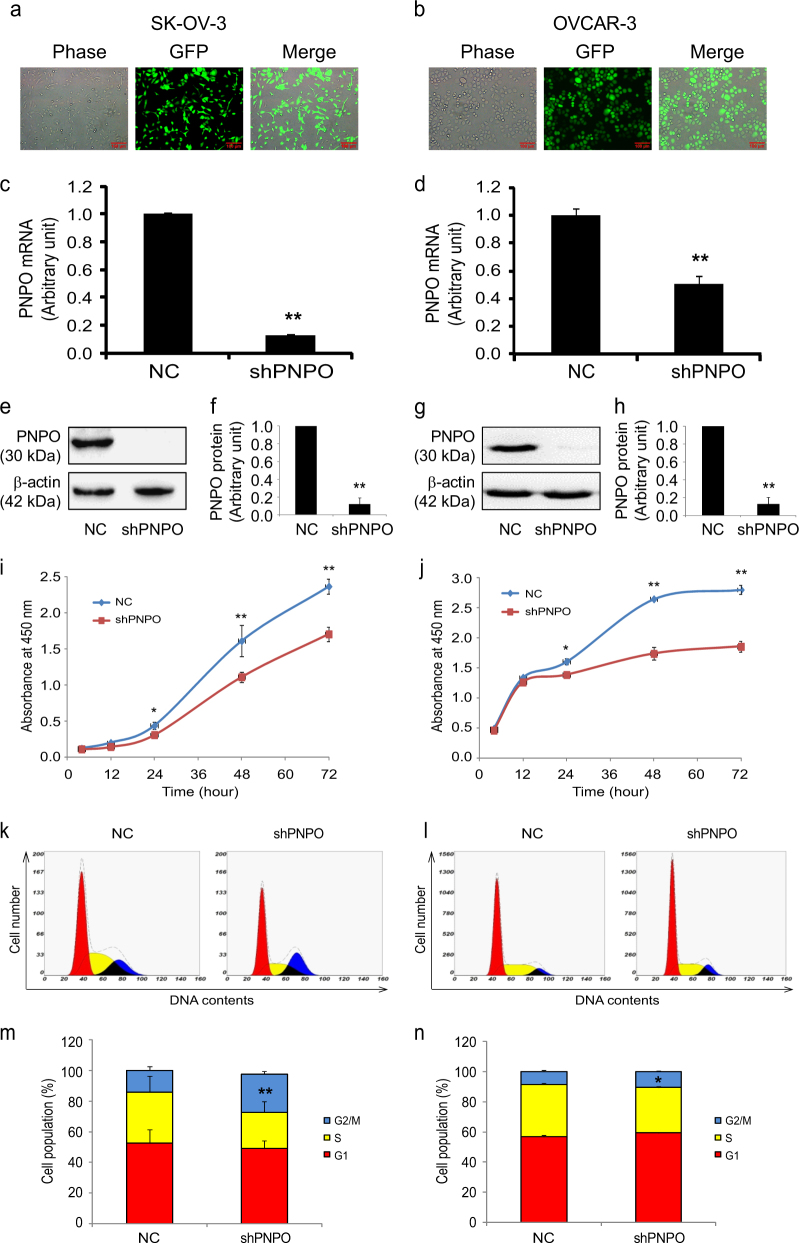
Effect of PNPO-shRNA on cell proliferation and cell cycle SK-OV-3 (**a**, **c**, **e**, **f**, **i**, **k** and **m**) and OVCAR-3 (**b**, **d**, **g**, **h**, **j**, **l** and **n**) PNPO-shRNA-expressing cells (shPNPO) are generated. **a**, **b** The phase contrast and fluorescent images of shPNPO cells at fifth passage are shown. Approximately 99% cells were GFP-positive. **c**, **d** PNPO mRNA expression was lower in shPNPO cells than in negative control (NC) cells detected by quantitative qRT-PCR. **e**−**h** PNPO protein expression was decreased in shPNPO cells detected by western blot using a specific antibody against human PNPO. Histograms show the semi-quantitative analysis of the relative optical density of protein bands (each *n* = 3). **i** and **j** A time-course study shows the proliferation of NC and shPNPO cells detected by the WST-1 assay. **k** and **l** Cell cycle of NC and shPNPO was determined by flow cytometry. **m** and **n** Histograms show the percentage of the cell population in each phase. *P < 0.05; **P < 0.01; *n* = 3 independent experiments

PNPO-shRNA constructs contained green fluorescent protein (GFP) and the expression of GFP in PNPO-shRNA-expressing cells was relatively stable in SK-OV-3 and OVCAR-3. Approximately 99% cells were GFP-positive without morphological change after five passages (Fig. 2a and b). Knockdown of PNPO was confirmed by qRT-PCR (Fig. 2c and d) and western blot (Fig. 2 e−h). Compared with control cells (NC), a significant decrease in cell growth was observed in PNPO-shRNA-expressing cells (shPNPO) at 24, 48 and 72 h post-seeding (*P* < 0.05, Fig. 2 i and j).

Flow cytometry revealed that the population of SK-OV-3 NC and shPNPO cells was 52.6 and 49.0% in G1 phase, 33.3 and 23.4% in S phase and 14.2 and 25.1% in G2/M phase, respectively (Fig. 2 k and m), whereas the population of OVCAR-3 NC and shPNPO cells was 56.7 and 59.3% in G1 phase, 34.8 and 30.1% in S phase and 8.5 and 10.6% in G2/M phase, respectively (Fig. 2 l and n). These data indicate that the knockdown of PNPO induces EOC cell cycle arrest at G2/M phase.

### Knockdown of PNPO inhibits migration and invasion of ovarian cancer cells

**Fig. 3:**
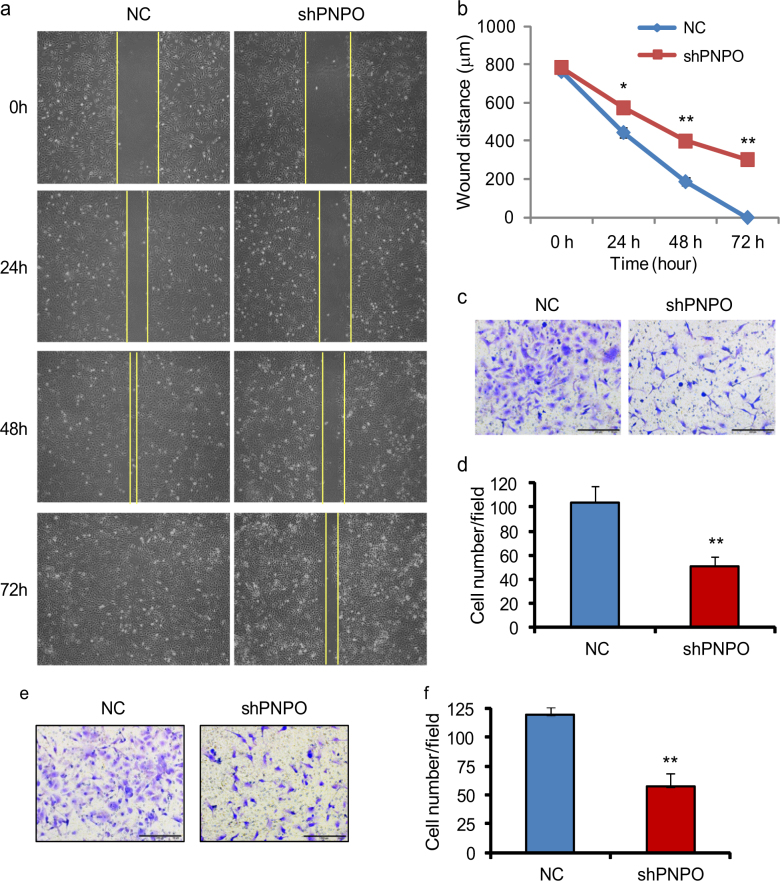
Effect of PNPO-shRNA on cell migration and invasion SK-OV-3 PNPO-shRNA-expressing cells (shPNPO) and negative control cells (NC) were used in experiments. **a** Wound healing assay. Photos of the wound were taken at 0, 24, 48 and 72 h after scratching. Original amplification, ×40. **b** Line graph shows the quantitative analysis of the wound healing at different time point. **c** Transwell assay of cell migration. Photos were taken at 48 h after seeding. Original amplification, ×200; scale bar, 50 µm. **d** Histogram shows the quantitative analysis of migrated cells of **c**, **e** Cell invasion assay. Photos were taken at 48 h after seeding. Original amplification, ×200; scale bar, 50 µm. **f** Histogram shows the quantitative analysis of invaded cells of **e**. Data are presented as mean ± SEM. *P < 0.05; **P < 0.01; *n* = 3 independent experiments

PNPO suppression significantly inhibited wound healing in SK-OV-3 PNPO-shRNA-expressing (shPNPO) cells compared with NC cells after monolayer scratch (Fig. 3 a and b). Further, migration assays using Transwell revealed that the number of migrating cells was reduced to 49% in shPNPO cells compared with NC cells at 48 h after seeding (Fig. 3 c and d). A similar result was found in OVCAR-3 PNPO-shRNA-expressing cells (Supplementary Figure 5a and 5b).

We also observed a reduced number of invading cells in SK-OV-3 PNPO-shRNA expressing (shPNPO) cells compared to NC cells. The number of invading cells was reduced to 52% in shPNPO cells compared with NC cells at 48 h after seeding (Fig. 3 e and f). Again, a similar result was found in OVCAR-3 PNPO-shRNA expressing cells (Supplementary Figure 5c and 5d). Collectively, these data indicate that the knockdown of PNPO inhibits cell migration and invasion.

### Knockdown of PNPO promotes ovarian cancer cell apoptosis

**Fig. 4:**
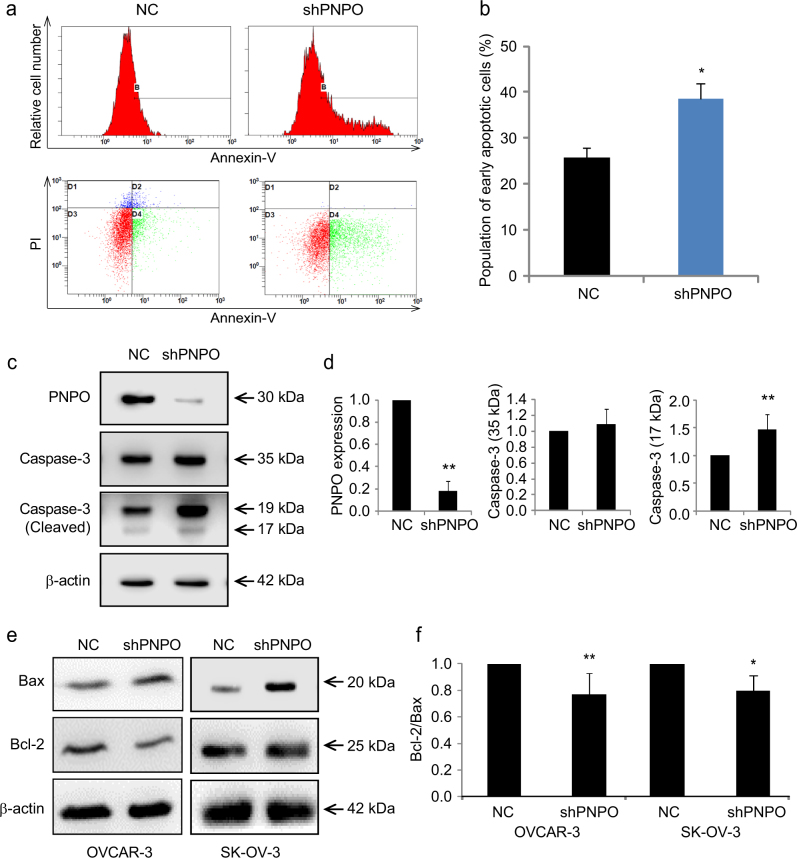
Effect of PNPO-shRNA on cell apoptosis **a** The population of apoptotic cells of SK-OV-3 PNPO-shRNA-expressing cells (shPNPO) and negative control cells (NC) was determined by flow cytometry. **b** Histogram shows the quantitative analysis of the population of early apoptotic cells from **a**, **c** Caspase-3 and PNPO proteins in NC and shPNPO cells were detected by western blot. Cleaved caspase-3 was increased, while PNPO was knocked down, in SK-OV-3 shPNPO cells. **d** Semi-quantitative analysis of the relative optical density of protein bands in **c** (*n* = 3). **e** Bax and Bcl-2 proteins in NC or shPNPO-expressing OVCAR-3 and SK-OV-3 cells detected by western blot. Bax protein was increased, while Bcl-2 protein was decreased, in shPNPO cells compared with NC cells. **f** Semi-quantitative analysis of the relative optical density of protein bands in **e**. Data are presented as mean ± SEM. *P < 0.05; **P < 0.01; *n* = 3 independent experiments

Flow cytometry analysis revealed that the number of apoptotic cells was significantly increased in SK-OV-3 shPNPO cells compared with NC cells (*P* < 0.05, Fig. 4 a and b). SK-OV-3 shPNPO cells also had a high level of cleaved caspase-3 protein compared with NC cells (Fig. 4 c and d). Further, the expression of pro-apoptotic factor Bax was increased, while the expression of anti-apoptotic factor Bcl-2 was decreased, in shPNPO cells compared with NC cells (Fig. 4e). The ratio of Bcl-2/Bax was significantly reduced (*P* < 0.05, Fig. 4f), confirming the induction of apoptosis after PNPO knockdown.

### Knockdown of PNPO suppresses tumour growth in an animal xenograft model

**Fig. 5:**
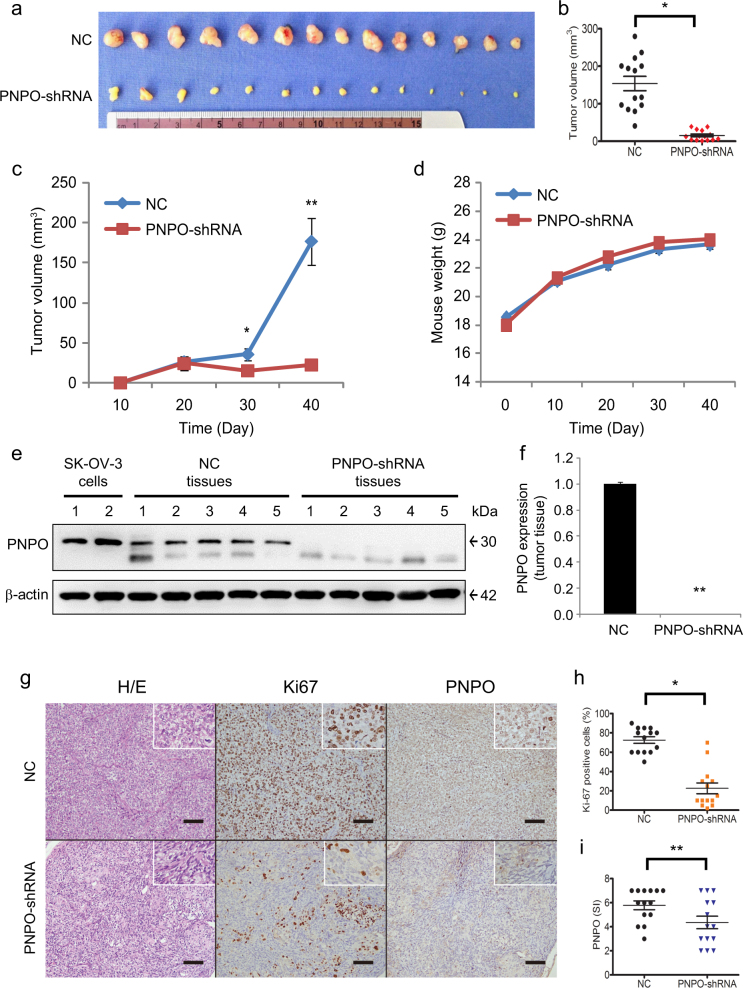
Suppression of tumour growth after knockdown of PNPO in nude mice Mice were implanted with either PNPO-shRNA-expressing cells (PNPO-shRNA) or negative control cells (NC) of SK-OV-3 and live for 40 days. **a** Photograph of excised tumour mass after the killing (*n* = 14). **b** Quantitative analysis of individual tumour volume in PNPO-shRNA and NC xenografts. **c** Live measurement of tumour volume in a time-course study. **d** Live measurement of mouse weight in a time-course study. **e** PNPO protein expression in tumour mass detected by western blot using a specific antibody against human PNPO. Arabic numerals represent the case number. The parental SK-OV-3 cells were used as positive control of detection (*n* = 2). **f** Semi-quantitative analysis of the relative optical density of protein bands in **e**. Data are presented as mean ± SEM (*n* = 5). **g** Tumour tissue sections were subjected to IHC for analysis of Ki-67 and PNPO expression. Representative images of Ki67 and PNPO expression in PNPO-shRNA and NC cells are shown. Original amplification, ×200, ×400 (insert). Scale bar, 100 µm. **h** Quantitative analysis of the percentage of Ki-67-positive cells. **i** Quantitative analysis of the percentage of PNPO-positive cells. *P < 0.05; **P < 0.01

Tumour volume from PNPO-shRNA mice was smaller than tumour volume from control SK-OV-3 (NC, control) mice (Fig. 5a). The average of tumour volume in control mice was about 150 mm^3^, whereas the tumour volume in PNPO-shRNA mice was less than 50 mm^3^ (*P* < 0.05, Fig. 5b). Tumour volume significantly increased in control mice compared to PNPO-shRNA mice by day 30 post-injection, persisting to the end of observation at day 40 (*P* < 0.05, Fig. 5c). Despite the difference in tumour volume, mouse body weight between the two groups was not different (Fig. 5d). Additionally, PNPO protein expression was almost undetectable in PNPO-shRNA tumours compared with control tumours (*P* < 0.01, Fig. 5 e and f), consistent with the low levels of immunoreactive staining of PNPO protein in PNPO-shRNA tumour sections (Fig. 5g). Moreover, quantitative analysis of proliferative marker Ki67 and PNPO-positive cells showed a significant decrease in the number of Ki-67-positive cells and PNPO-shRNA-expressing cells after PNPO knockdown (*P* < 0.05, Fig. 5 h and i). These data indicate that PNPO affects tumour growth and cell proliferation and may influence ovarian tumorigenesis.

### PNPO expression and cell proliferation are influenced by vitamin B6 vitamers

**Fig. 6:**
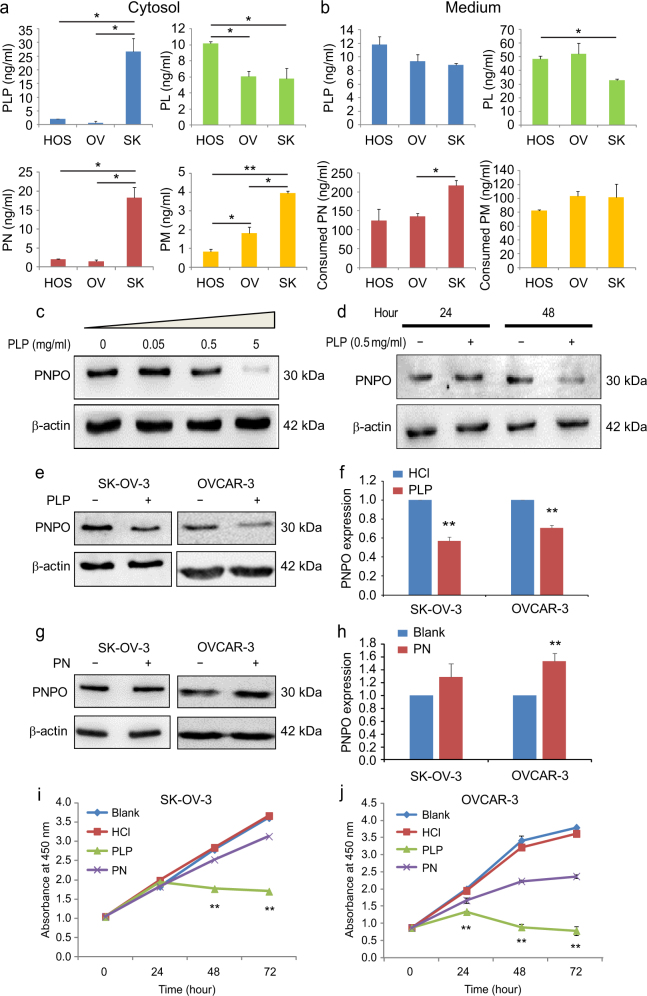
Effect of vitamin B6 on PNPO expression and cell proliferation **a**, **b** Concentration of vitamin B6 vitamers (PLP, PL, PN and PM) in the cytosol and culture medium of three epithelial ovarian cell lines HOSEpiC (HOS), OVCAR-3 (OV) and SK-OV-3 (SK). **c** Dose-dependent effect of PLP on PNPO protein expression in SK-OV-3 cells. Cells were treated with 0, 0.05, 0.5 and 5 mg/ml of PLP for 48 h. PNPO protein expression was detected by western blot. **d** Time-course effect of PLP on PNPO protein expression in SK-OV-3 cells. Cells were treated with 0.5 mg/ml of PLP for 24 and 48 h. PNPO protein expression was detected by western blot. **e** PNPO protein expression in SK-OV-3 and OVCAR-3 cells after 0.5 mg/ml of PLP treatment for 48 h. **f** Semi-quantitative analysis of the relative optical density (ROD) of protein bands in **e**, **g** PNPO expression in SK-OV-3 and OVCAR-3 cells after 0.5 mg/ml of PN treatment for 48 h. **h** Semi-quantitative analysis of the ROD of protein bands in **g**, **i**, **j** Measurement of cell proliferation. SK-OV-3 and OVCAR-3 cells were treated with PLP, PN or vehicle HCl for 24, 48 and 72 h. Untreated cells were used as control (blank). Cell proliferation was detected by the WST-1 assay. Data are presented as mean ± SEM. *P < 0.05; **P < 0.01; *n* = 3 independent experiments

To evaluate the enzymatic activity of PNPO, we first measured the concentrations of PN, PM, PLP and PL vitamers in the cytosol and culture medium. The basal level of cytosolic PN, PM and PLP was higher in SK-OV-3 cells compared with OVCAR-3 and HOSEpiC cells (Fig. 6a). Because PN and PM are essential for living cells during culture, a commercial culture medium must contain PN and PM. Thus, the amount of consumed PN and PM were calculated by the concentrations of PN and PM in the original medium minus the concentrations of that measured in the cultured medium. We found that SK-OV-3 cells consumed more PN and PM from culture media after 48 h incubation and produced more cytosolic PLP (*P* < 0.05, Fig. 6b). These data indicate that SK-OV-3 cells may have more PNPO activity, which is consistent with the high levels of PNPO protein expression in SK-OV-3 cells compared with OVCAR-3 and HOSEpiC cells (Supplementary Figure S3).

Treatment of SK-OV-3 cells with PLP and PN revealed that PLP suppressed PNPO expression in time- and dose-dependent manners (Fig. 6 c and d). Administration of PLP (0.5 mg/ml) downregulated, whereas administration of PN (0.5 mg/ml) upregulated PNPO protein expression in SK-OV-3 and OVCAR-3 cells (Fig. 6 e−h), indicating a feedback loop between PNPO and vitamers.

Subsequently, we investigated the extent to which PLP influences cell proliferation and found that compared with control groups (Blank and HCl vehicle), SK-OV-3 cells at 48 and 72 h (*P* < 0.01, Fig. 6i) and OVCAR-3 cells at 24, 48 and 72 h (*P* < 0.01, Fig. 6j) had reduced cell growth after PLP administration. We also observed a decrease in cell proliferation in both SK-OV-3 and OVCAR-3 cells at 48 and 72 h after PN administration (*P* < 0.01, Fig. 6 i and j). Higher PN may stimulate PNPO expression, which in turn converts it into PLP, whereas an increase in PLP negatively regulates PNPO expression, with downstream impact on cell proliferation. These data further confirm that there is a feedback loop between PNPO and vitamers.

### PNPO expression is regulated by the TGF-β signalling pathway via miR-143-3p

**Fig. 7:**
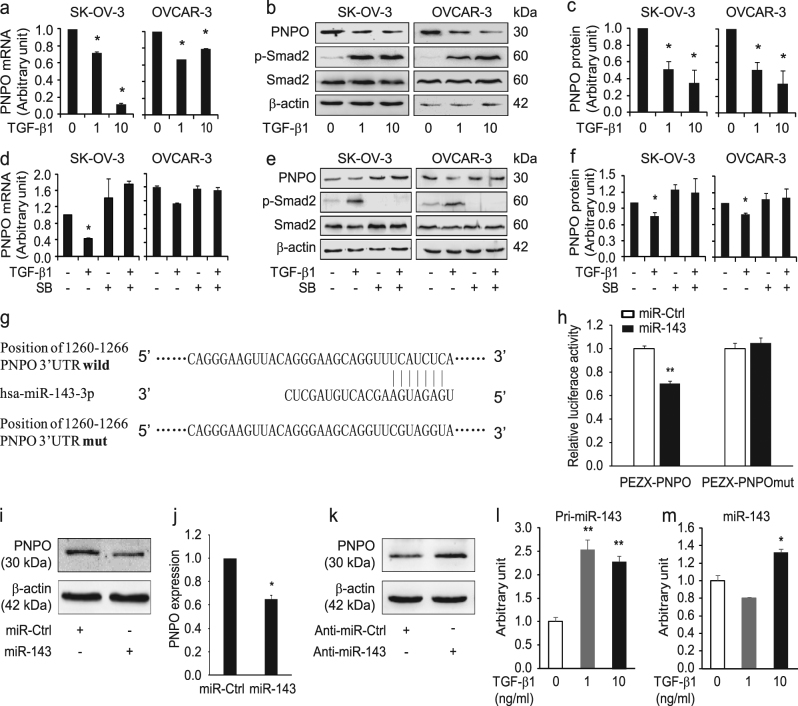
Regulation of PNPO expression by the TGF-β signalling pathway **a**, **b**, **c** Effect of TGF-β1 on PNPO expression. SK-OV-3 and OVCAR-3 cells were treated with TGF-β1 at different concentrations (0, 1, 10 ng/ml). **a** PNPO mRNA expression was detected after 24 h treatment. **b** PNPO protein expression was detected after 48 h treatment. **c** Semi-quantitative analysis of the relative optical density of protein bands in **b** (*n* = 3). **d**, **e**, **f** Effect of TGF-β type I receptor kinase inhibitor SB431542. Cells were pre-treated with 10 µm SB431542 for 30 min and then treated with 1 ng/ml of TGF-β1. **d** PNPO mRNA expression was detected after TGF-β1 treatment for 24 h. **e** PNPO protein expression was detected after TGF-β1 treatment for 48 h. **f** Semi-quantitative analysis of the relative optical density of protein bands in **e** (SK-OV-3, *n* = 5; OVCAR-3, *n* = 4). Phospho-Smad2 (p-Smad2) and β-actin were used as controls in western blot analysis. **g** A schematic diagram illustrated the sequences of the putative miR-143-3p binding site in the PNPO 3′-UTR and its mutant. **h** Dual-luciferase reporter assay. The interaction of miR-143-3p with the PNPO mRNA at 3′-UTR in HEK293T cells was confirmed (*n* = 3). **i** Effect of miR-143 mimics (50 nm for 72 h) on PNPO protein expression detected by western blot in OVCAR-3 cells. **j** Semi-quantitative analysis of the relative optical density of protein bands in **I** (*n* = 3). **k** Effect of miR-143 inhibitor (anti-miR-143) (150 nm for 72 h) on PNPO protein expression detected by western blot in OVCAR-3 cells. **l** Effect of TGF-β1 (0, 1 and 10 ng/ml for 3 h) on primary miR-143 (Pri-miR-143) expression detected by qRT-PCR in OVCAR-3 cells. **m** Effect of TGF-β1 (0, 1 and 10 ng/ml for 3 h) on mature miR-143 expression detected by qRT-PCR in OVCAR-3 cells. Data are presented as mean ± SEM. *P < 0.05; **P < 0.01

Since TGF-β plays a key role in ovarian tumorigenesis and PNPO is overexpressed in OC, we next investigated the influence of TGF-β signalling on the PNPO expression in two EOC cells (SK-OV-3 and OVCAR-3). PNPO expression in SK-OV-3 and OVCAR-3 cells was significantly decreased after TGF-β1 treatment both at the mRNA (Fig. 7a) and protein (Fig. 7 b and c) levels (*P* < 0.05). Because Smad2 is a transducer protein and is activated upon TGF-β1 treatment, the increased phosphorylation of Smad2 can be considered as a cell’s responsiveness to TGF-β1. Indeed, p-Smad2 was increased in SK-OV-3 and OVCAR-3 cells after TGF-β1 treatment (1 and 10 ng/ml) for 24 h (Fig. 7b).

By blocking the TGF-β signalling pathway using a TGF-β type I receptor kinase inhibitor SB-431542, we found that the effect of TGF-β1 on PNPO expression at the mRNA and protein levels was abolished (Fig. 7 d−f). Interestingly, a decrease in PLP levels in the cytosol and an increase in PLP levels in the media were observed in SK-OV-3 cells after 10 ng/ml TGF-β1 treatment (Supplementary Figure S6), suggesting that TGF-β1 may stimulate the PLP secretion. These actions of TGF-β1 were also abolished by SB-431542 (Supplementary Figure S6), indicating that PNPO expression and vitamin B6 metabolism are mediated by the TGF-β signalling pathway.

Using miRWalk 2.0 database^33^, we found that PNPO is a potential target of miR-143-3p. In order to confirm the interaction of miR-143-3p with PNPO mRNA, we constructed two plasmids: a wild-type PNPO 3′-UTR containing a miR-143-3p binding site (position at 1260−1266 of 3′-UTR) and a mutated PNPO 3′-UTR in which the binding site was changed (Fig. 7g). Dual-luciferase assay confirmed that wild-type 3′-UTR, not mutated 3′-UTR, bound to PNPO 3′-UTR (Fig. 7h). Treating SK-OV-3 cells with miR-143 mimics resulted in decreased PNPO protein expression (*P* < 0.05; Fig. 7 i and j), whereas treating cells with miR-143 inhibitor (anti-miR-143) resulted in increased PNPO protein expression (Fig. 7k). Notably, TGF-β1 enhanced primary and mature miR-143-3p expression (*P* < 0.05; Fig. 7 l and m). These data suggest that TGF-β-mediated PNPO expression is at least in part controlled through the upregulation of miR-143-3p.

To determine the correlation between PNPO mRNA expression and miR-143 expression in patients with ovarian tumours, we performed a qRT-PCR in ovarian tissues of 14 patients (4 benign, 3 borderline, 7 malignant tumours) and 5 normal controls. We found that there was a slight negative-correlation in ovarian tissues (*n* = 19 individuals, Supplementary Figure S7). The high level of PNPO mRNA expression was also found in serous (*n* = 41), mucinous (13) and endometrioid (*n* = 37) adenocarcinoma as well as in clear cell carcinoma (*n* = 8) compared with normal ovary (*n* = 4) in the microarray data sets of Hendrix from Oncomine database (www.oncomine.org) (Supplementary Figure S8). These data further confirm the overexpression of PNPO in OC, indicating that PNPO may play a role in OC development.

Next, we examined the effect of PNPO-shRNA on PLP concentration and Smad2 phosphorylation in EOC cells. We found that knockdown of PNPO resulted in a decrease of PM, PLP and PL levels in the cytosol (*P* < 0.05, Supplementary Figure S9a), a reduction of PL level in medium of SK-OV-3 (*P* < 0.01, Supplementary Figure S4b), and an increase of PLP level in the medium of OVCAR-3 (*P* < 0.01, Supplementary Figure S9b). Silencing of PNPO after PNPO-shRNA infection was confirmed by western blot in OVCAR-3 and SK-OV-3 cells (Supplementary Figure S9c and S9d). Interestingly, we also observed an increase of Smad2 phosphorylation after PNPO knockdown (*P* < 0.05, Supplementary Figure S9c and S9d), suggesting that there is a negative feedback loop between PNPO and TGF-β signalling.

## Discussion

Our study provides new insights into the role and function of PNPO in OC. We demonstrated for the first time that PNPO is overexpressed in EOC. Knockdown of PNPO affects EOC cell behaviour in vitro and influences tumour formation in vivo. Furthermore, PNPO is mediated by the TGF-β signalling pathway through the upregulation of miR-143-3p.

Most studies on PNPO have been conducted in the context of epilepsy development. Deficiency or mutation in PNPO is known to be a cause for neonatal and infantile epileptic encephalopathy^15,34,35^. However, the role of PNPO and the mechanisms underlying the regulation of PNPO in cancer progression, especially in EOC, are unknown. Our previously unpublished data in a screening study identifying early detection biomarkers showed several molecules involved in OC development, including PNPO. Currently, only three publications exist regarding a relationship between PNPO and cancer. The first report was published in 1998 describing the absence of PNPO activity in liver and brain tumours^20^. Nine years later in 2006, another report showed that PNPO is induced by estradiol and 4-hydroxytamoxifen in T47D and MCF7 breast cancer cells, but not in ERα-negative MDA-MB-231 breast cancer cells^19^. During the preparation of this manuscript, a new study reported that PNPO is one of seven genes that can predict overall survival of patients with colorectal cancer^21^. These data suggest that PNPO may be implicated in tumorigenesis. Yet, none of these studies explored the mechanisms of how PNPO is regulated in cancer.

Our study provides the evidence of PNPO overexpression in the tumour tissues of human EOC, including serous, mucinous, endometrioid, transitional cell and clear cell carcinoma. The expression of PNPO protein was significantly correlated with age, histological type and grade. Higher levels of PNPO expression were found in surface epithelial malignant tumours rather than sex cord-stromal and germ cell tumours, indicating that the overexpression of PNPO may be EOC specific. Furthermore, PNPO expression was increased with age and grade, suggesting that PNPO may play a role in EOC development. Our data are consistent with a cohort of data sets found in Oncomine, which PNPO mRNA was higher in serous, mucinous, endometrioid and clear cell carcinomas compared with the normal ovary. These data further confirm the overexpression of PNPO in OC, indicating that PNPO may play a role in OC development. In the future, nevertheless, it may be of interest to examine PNPO expression in patients following treatment and to measure secreted PNPO in the serum and urine of patients to see if PNPO as a biomarker of EOC can be detected in body fluids.

The function of PNPO in cancer, especially in EOC, had not been reported before. Using a loss-of-function approach we examined the biological functions of PNPO in vitro and in vivo, specifically apoptosis, proliferation, migration, invasion and colony formation in EOC cells. Knockdown of PNPO induced cell apoptosis and decreased cell proliferation, migration and invasion. Furthermore, silencing of PNPO inhibited tumour formation in the orthotopically implanted nude mice. These data, along with the observation of PNPO overexpression in human tumour tissues, suggest that PNPO may contribute to OC progression.

In vitamin B6 metabolism, PN and PM from food and dietary supplements are precursors of PNP and PMP that are oxidized into PLP by PNPO and finally metabolized into PL^36^. PLP is an active form of vitamin B6 and it has been estimated that about 4% of all enzyme activities use PLP as a cofactor^37^. Our study unveiled that increased PLP can suppress PNPO protein expression, resulting in the inhibition of EOC cell proliferation. These data may suggest that PLP is important for the regulation of PNPO expression, in addition to it being a product of PNP/PMP oxidation by PNPO. A feedback loop between PLP and PNPO may exist, but the mechanism how PLP regulates PNPO expression remains unclear and thus requires further study.

Increasing evidence shows that TGF-β signalling plays an important role in the development of OC^38,39^. Alteration of TGF-β signalling is crucial for ovarian tumorigenesis^40^. Our previous studies have shown that TGF-β1 regulates cystatin B^30^, β-2-microglobulin^29^ and CMPK1^31^, three progression markers of the human epithelial ovarian tumour. The current study also demonstrated that PNPO is another EOC progression marker regulated by the TGF-β signalling pathway. We found that TGF-β1 upregulated miR-143-3p and that PNPO was a target gene of miR-143-3p. These data suggest that the downregulation of PNPO expression by TGF-β1 is at least in part via the upregulation of miR-143-3p. We speculate that the loss of TGF-β responsiveness or a defect in TGF-β signalling may lead to the downregulation of miR-143-3p and, in turn, the upregulation of PNPO. Interestingly, we found that knockdown of PNPO increased the phosphorylation of Smad2, most likely due to the negative feedback loop between PNPO and TGF-β signalling.

The current study utilized clinical samples that showed a slight negative-correlation between PNPO and its regulator miR-143. It has been shown that a chemically modified miR-143 significantly suppresses the tumour formation in a colorectal cancer model^41^. Thus, we speculate that PNPO and its regulators are the candidates of a potential target for the treatment of EOC.

**Fig. 8:**
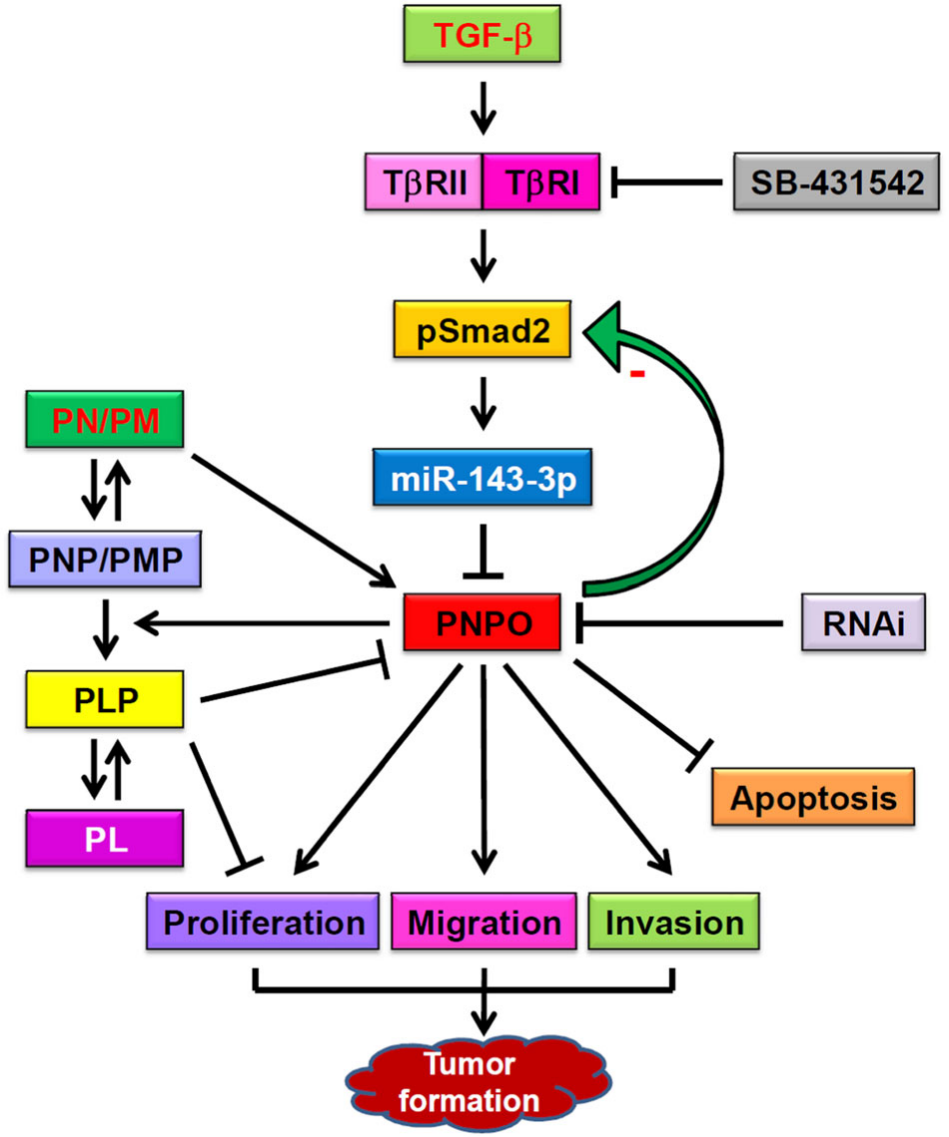
Schematic illustration of the mechanism of PNPO regulation and the intracellular conversion of vitamin B6 vitamers in ovarian cancer cells TGF-β1 binds to its type II receptor and recruits type I receptor to form a receptor complex, which then phosphorylates and activates intracellular transducer protein Smad2. MiR-143-3p is upregulated upon TGF-β1 stimulation and interacts with the 3′-UTR of PNPO mRNA, leading to a downregulation of PNPO expression. Blocking type I receptor by SB-431542 abolishes the TGF-β action. PNPO is upregulated in epithelial ovarian cancer cells. Knockdown of PNPO by RNA interference results in the inhibition of cell proliferation, migration and invasion, the induction of apoptosis, the retard of tumour formation and a negative feedback loop with Smad2 phosphorylation. Dietary pyridoxine (PN) and pyridoxamine (PM) serve as the main source of pyridoxine 5′-phosphate (PNP) and pyridoxamine phosphate (PMP). PNP and PMP are then converted to pyridoxal 5′-phosphate (PLP), an active form of vitamin B6, by the catalysing enzyme - pyridoxine 5′-phosphate oxidase (PNPO). PLP is finally hydrolysed to pyridoxal (PL). Increased PLP inhibits PNPO expression and cell proliferation

In summary, we have demonstrated for the first time that PNPO is overexpressed in human EOC, suggesting that PNPO may be a useful tissue biomarker of OC. PNPO is downregulated by the TGF-β signalling pathway through the upregulation of miR-143-3p. Silencing of PNPO induces OC cell apoptosis and decreases cell proliferation, migration and invasion in vitro and tumour formation in vivo. A schematic illustration of the mechanisms of PNPO regulation and intracellular conversion of vitamin B6 vitamers is shown in Fig. 8. Based on the fact that decreased PNPO resulted in a change of cell behaviour, we predict that PNPO may be a potential diagnostic biomarker and therapeutic target in patients with EOC. Inhibition of PNPO by the administration of miRNA, RNAi and PLP may be a good therapeutic strategy against OC.

## Methods

### Patients and ovarian tissue samples

Informed consent was obtained from patients and ethics approval was obtained from the Ethics Committee of Jinshan Hospital, Fudan University. Cytoreductive surgery was performed on 73 patients at Jinshan Hospital from January 2005 to December 2013. Patients had not received chemotherapy or radiotherapy before surgery. Ovarian tissue samples were obtained from patients with ovarian tumour (24 benign cases, 18 borderline cases and 31 malignant cases). Control ovarian tissues were obtained from nine patients with non-tumorous ovaries. Ovarian tissues were formalin-fixed, paraffin embedded or frozen for subsequent histological and biochemistry analyses, respectively.

### Pathology assessment

Formalin-fixed, paraffin embedded ovarian tissue specimens underwent pathological examination in the Department of Pathology, Jinshan Hospital. Histological grades and stages were classified based on the current criteria of tumour, node and metastasis (TNM) classification from the American Joint Committee on Cancer (AJCC) and the World Health Organization (WHO). The final diagnosis of disease was made by experienced gynaecologists and pathologists according to the FIGO (International Federation of Gynaecological Oncologists) system.

### Tissue microarray

Ovarian tissue microarrays (Cat# OV20813 and Cat# OC 20814, Xi’an Alena Biotechnology Co. Ltd., Xi’an, China) were used for the IHC analysis. A total of 416 paraffin-embedded ovarian tissue samples from individuals with a median age of 48 years (range 11–88 years) were collected and of these, 404 samples were valid (388 malignant tumours, 7 normal ovarian tissues and 9 adjacent normal tissues of OC) and 12 samples were either invalid or missing after IHC. Clinical characteristics including age, histological type, grade, stage and lymph node metastasis were recorded.

### Immunohistochemical staining and analysis of human ovarian tissue

Immunohistochemistry was performed as described previously^30^. After blocking with 10% normal goat serum (Maixin Bio, Fuzhou, Fujian, China) for 40 min at room temperature, sections were incubated at 4 °C overnight with a monoclonal mouse anti-PNPO antibody (1:300 dilution, Abcam, New Territories, Hong Kong, China), followed by incubation at room temperature for 1 h with biotinylated anti-mouse secondary antibody (Maixin Bio). The immunoreactive signal was detected using a DAB Kit (Maixin Bio). Sections were then counterstained with hematoxylin and photographed under a light microscope (BX43, OLYMPUS, Tokyo, Japan) at ×100 and ×400 magnification. Brown colour staining in the cell was considered as PNPO-positive staining. Evaluation of immunoreactive staining was performed by two pathologists without any prior knowledge of the patient’s clinical data. Pathologists then resolved initial discordant staining scores through discussion and consensus. A staining index (SI)^30^ was determined by the sum of the positive extent and staining intensity and expressed as PNPO-negative and PNPO-positive. In brief, the percentage of PNPO immuno-positive cells were scored as 0 (no positive cell), 1 (⩽25% positive cells), 2 (26–50% positive cells), 3 (51–75% positive cells) and 4 (>75% positive cells). The intensity of immunoreactive staining was also scored as 0 (no staining), 1 (weak staining), 2 (moderate staining) and 3 (strong staining). A final immunoreactive score, referred to SI, was determined by the sum of the immuno-positive cells and staining intensity, and was clustered into four groups: '0', ⩽2 sum points; '1', 3–4 sum points; '2', 5–6 sum points; and '3', 7 sum points. Cases with a final SI score of 0 and 1 were classified as PNPO-negative and the cases with a final SI score of 2 and 3 were classified as PNPO-positive.

### Cell culture

Human OC cell lines OVCAR-3 (adenocarcinoma), SK-OV-3 (adenocarcinoma), CAOV-3 (adenocarcinoma) and ES-2 (clear cell carcinoma) cells were purchased from American Type Culture Collection (ATCC, Manassas, VA, USA). Human ovarian carcinoma cell line A2780 was purchased from the European Collection of Authenticated Cell Cultures (ECACC, UK). OVCAR-3 and A2780 cells were cultured in RPMI-1640 and SK-OV-3 and CAOV-3 cells were cultured in Dulbecco's Modified Eagle's Medium (DMEM, 4.5 g/L glucose, Corning Inc., Manassas, VA, USA) supplemented with 10% fetal bovine serum (FBS, Invitrogen, Carlsbad, CA, USA). ES-2 cells were cultured in McCoy’s 5A medium (Sigma, Saint Louis, MO, USA) supplemented with 10% FBS. Non-tumorous human ovarian surface epithelial cells (HOSEpiC) as control cells were obtained from Guangzhou Jennio Biotech Co., Ltd (Guangzhou, Guangdong, China) and cultured in RPMI-1640 medium supplemented with 10% FBS. Dulbecco's Modified Eagle's Medium (DMEM), ethylenediaminetetraacetic acid (EDTA),phenylmethylsulfonyl fluoride (PMSF)

### PNPO-siRNA transfection

For transient transfection of siRNA, cells were first seeded in a six-well plate at a density of 2×10^5^ (OVCAR-3 cells) or 1.5×10^5^ (SK-OV-3 cells) per well for 24 h. In order to determine the knockdown efficiency of PNPO by PNPO-specific short interfering RNA (siRNA) for subsequent experiments, cells were transiently transfected with three different PNPO-siRNAs and an NC siRNA (GenePharma Co., Ltd., Shanghai, China) (Supplementary Table S2), respectively, at a final concentration of 2 µg/well for 6 h using X-tremeGENE siRNA Transfection Reagent (Roche Diagnostics, Indianapolis, IN, USA) as described by the manufacturer’s instructions, followed by western blot.

### Generation of PNPO-shRNA construct and PNPO-shRNA expressing cells

Human PNPO short hairpin RNA (PNPO-shRNA, shPNPO) was constructed with double-strand oligonucleotides corresponding to the target sequence of GACTGGCTCTATGAGAGAC and inserted into pHY-LV-KD5.1 RNAi lentivirus (Hanyin Biotechnology Co., Ltd., Shanghai, China). The sense and antisense sequences of PNPO-shRNA are listed in Supplementary Table S2.

To generate PNPO-shRNA-expressing cells and their counterpart control cells, OVCAR-3 and SK-OV-3 cells were seeded in six-well plates and infected with PNPO-shRNA or control lentiviral particles at a concentration of 15 and 20 multiplicity of infection, respectively, with polybrene at a final concentration of 8 µg/ml. After incubation for 24 h, cells were washed and cultured with medium supplemented with 3 µg/ml of puromycin (to kill uninfected cells). The efficiency of PNPO-shRNA lentiviral transduction was examined by fluorescence microscopy as the constructs contained GFP. Knockdown of PNPO was confirmed by qRT-PCR and western blot. The fourth or fifth passaged cells that stably expressed PNPO-shRNA were used for cellular assays.

### RNA extraction and quantitative real-time PCR

Total RNA was extracted from tissues and cells using Trizol (Invitrogen) according to the manufacturer’s instruction and as described previously^30^. PCR primer sequences (Supplementary Table S2) were synthesized by Sangon Biotech Co., Ltd. (Shanghai, China). Reaction conditions of cDNA synthesis were as 30 °C for 10 min, 42 °C for 50 min, 85 °C for 5 min, 4 °C for 60 min. PCR amplification was performed at 95 °C for 10 min, followed by 40 cycles of 95 °C for 10 s and 60°C for 31 s using an SYBR Green Master kit (Roche Applied Science). Samples were run in triplicate and experiments were repeated at least three times. The expression level of PNPO mRNA normalized to an endogenous control β-actin or target miRNA normalized to its control U6 was calculated using 2^ΔΔCt^, in which threshold cycle (Ct) was obtained using Sequence Detection Software V1.4 (7300 Real Time PCR System, Applied Biosystems, Foster City, CA, USA).

### Protein extraction and western blot analysis

PNPO-shRNA and control cells and ovarian tissues were lysed in sodium dodecyl sulfate (SDS) lysis buffer (Beyotime, Haimen, Jiangsu, China) with 1% phenylmethylsulfonyl fluoride (PMSF, Beyotime) and 1% phosphatase inhibitor (Key-GEN), followed by sonication and SDS-polyacrylamide gel electrophoresis. The following primary antibodies were used: mouse anti-PNPO (1:4000 dilution, Abcam), rabbit anti-Bcl-2 (1:1000 dilution, Santa Cruz), mouse anti-Smad 2 (1:2000 dilution), rabbit anti-phospho-Smad2 (1:1000 dilution), rabbit caspase-3 (1:1000 dilution), rabbit anti-Bax (1:1000 dilution) and rabbit anti-β-actin (1:5000 dilution) (Cell Signaling Technology, Inc., Danvers, MA, USA). Secondary antibodies were horseradish peroxidase-conjugated goat anti-rabbit IgG and anti-mouse IgG (1:10,000 dilution, Cell Signaling Technology, Inc.). Signals were detected using Immobilon^TM^ Western Chemiluminescent HRP Substrate (Millipore) and quantified using Tanon-4500 Gel Imaging System with GIS ID Analysis Software v4.1.5 (Tanon Science & Technology Co., Ltd., Shanghai, China).

### Cell proliferation and cell cycle progression assays

Control and PNPO-shRNA-expressing cells were seeded in 96-well plate at a density of 3×10^3^/well (SK-OV-3) or 5×10^3^/well (OVCAR-3) and cultured for 24, 48 or 72 h. Cell proliferation was detected by the WST-1 assay (Roche Diagnostics, Indianapolis, IN, USA) according to the manufacturer’s instruction.

Cell cycle was measured by flow cytometry. In brief, cells were seeded in six-well plates and cultured for 48 h. After trypsinization, the cells were washed with cold phosphate buffered saline (PBS) twice at 1000 rpm for 5 min each and fixed with cold 70% ethanol. After washing cells with PBS three times, the pellet was resuspended in 500 µl of propidium iodide (PI) solution (PI/RNase Staining Buffer, BD Pharmingen, San Diego, CA, USA) and incubated in the dark at room temperature for 15 min. Ten thousand cells were acquired by flow cytometry (Beckman Coulter, Inc., Brea, CA, USA). Flow data were analysed using Wincycle software (Beckman Coulter) and presented as the percentage of the cell population in G0/G1, S and G2/M phases.phosphate buffered saline (PBS)

### Migration assay

To assess whether PNPO silencing impacts cell migration, wound healing and transwell migration assays were conducted. To measure the wound-fill rate in vitro, PNPO-shRNA-expressing cells and control cells were first cultured, respectively, in six-well plates and grew until subconfluence. An approximately 800-μm-wide wound in the plate was created by scraping the monolayer with a sterile 200-µl pipette tip. The width of the gap in the monolayer was photographed under a microscope and measured using CellSens Life Science Imaging Software (OLYMPUS).

Transwell migration assay was performed in a 24-well transwell chamber with a polycarbonate membrane (6.5 mm in diameter with 8-μm pores, Costar Corning, New York, NY, USA). In brief, cells were plated into the upper chamber at a density of 5×10^3^ cells/well with 800 µl of medium without serum. The lower chamber was filled with 600 µl of medium containing 10% FBS. After incubation for 24 or 48 h, cells that had not migrated to the bottom chamber were gently removed from the upper chamber by a swab. Cells that migrated through the membrane were fixed with 4% paraformaldehyde, stained with 5% Crystal Violet Staining Solution (Beyotime), and photographed under a light microscope (OLYMPUS) at ×400 magnification.

### Invasion assay

The extent of invasive migration of PNPO-silenced cells was assessed using a standard invasion assay. Matrigel (final concentration 250 µg/ml, BD Biosciences #356234, Bedford, MA, USA) was coated on the top of a polycarbonate membrane before seeding the PNPO-shRNA-expressing and NC cells. The cells were plated into the upper chamber at a density of 5×10^3^ cells/well and cultured under the same conditions as described in the migration assay. After 24 or 48 h, cells that had not invaded the bottom chamber were gently removed from the upper chamber by a swab. Cells that invaded through the membrane were fixed, stained, photographed and counted as in migration assay.

### Detection of apoptosis

Apoptotic cells were detected by staining EOC cells with annexin-V conjugated with APC (Allophycocyanin, BD Pharmingen, BD Biosciences) and PI (BD Pharmingen). In brief, cultured cells were trypsinized (in the absence of ethylenediaminetetraacetic acid, EDTA) and washed twice with cold PBS. Cells were then resuspended in 1× binding buffer at a density of 1×10^6^cells/ml. After transferring 100 µl of cell suspension into a 5 ml tube, 5 µl of APC Annexin-V and/or 5 µl of PI was added. Cells were then incubated in the dark for 15 min at room temperature. After adding 400 µl of 1× binding buffer to each tube, the cell population was immediately measured by flow cytometry.

### Determination of PNPO activity

PNPO activity was determined by measuring vitamin B6 vitamers in PNPO-shRNA and control SK-OV-3 cell lysates and culture medium using Liquid Chromatogram (Waters®Xevo®TQ-Smicro, Milford, MA, USA). Standard curves of PLP, pyridoxine (PN), pyridoxamine (PM) and pyridoxal (PL) were generated using commercial products. Five microliters of standards and samples were respectively added into a liquid chromatogram. The concentrations of different vitamers in cell lysates and culture media were quantified and PNPO activity was indirectly determined by the amount of upstream and downstream vitamers.

### Treatment with TGF-β1 and its receptor inhibitor

After seeding for 24 h, PNPO-shRNA-expressing cells and control cells were treated with recombinant human TGF-β1 (R&D Systems, Minneapolis, MN, USA) at 0, 1 or 10 ng/ml for 24 or 48 h. To block TGF-β signalling, cells were pre-treated for 30 min with 10 µm SB-431542 (Sigma), a TGF-β type I receptor kinase inhibitor^42^, which was dissolved in dimethyl sulfoxide (DMSO) at a concentration of 10 mm, and then treated with TGF-β1 for 24 h (mRNA) or 48 h (protein). DMSO was used as a treatment control.

### Transfection of microRNA mimics/inhibitors and dual-luciferase reporter assay

After seeding at a density of 2×10^5^ cells/well into a six-well plate and incubation for 16 h, OVCAR-3 cells were transfected with 50 nm miR-143-3p mimics, 50 nm miR-negative control (miR-Ctrl), 150 nm miR-143-3p inhibitors (anti-miR-143) or 150 nm anti-miR-negative control (anti-miR-Ctrl) (Supplementary Table S2) (RiboBio Co., Ltd, Guangzhou, Guangdong, China) using X-tremeGENE Transfection Reagent (Roche Applied Science) and cultured for 72 h.

A partial 3′-UTR of PNPO (479 bp) that contains the predicted binding site of miR-143-3p was amplified from genomic DNA by using the Pfu Ultra II Fusion HS DNA Polymerase (Stratagene, Agilent Technologies, Santa Clara, CA, USA) with specific primers (Supplementary Table S2). The PCR product was inserted into a Duo-Luciferase reporter vector pEZX-FR2 (GeneCopoeia Inc, Rockville, MD, USA) after linearizing by *Eco*RI and *Xho*I restriction enzymes using EasyGeno Assembly Cloning kit (Tiangen Biotech Co., Ltd, Beijing, China), which was named as pEZX-PNPO. The 3′-UTR mutant pEZX-PNPOmut was generated in the consensus miR-143-3p binding site by using a QuikChange II site-directed mutagenesis kit (Stratagene). All constructs were verified by restriction enzyme digestion and DNA sequencing. To determine the expression of our clone, subconfluent HEK293T cells were cultured in 24-well plates and co-transfected with 0.5 µg pEZX-PNPO (or pEZX-PNPOmut) reporter plasmid and 50 nm miR mimics (or NCs) using Roche X-tremeGENE Transfection Reagent. After 24 h, cells were lysed and luciferase activities were determined using Luc-Pair™ Duo-Luciferase Assay Kit (GeneCopoeia Inc) according to the manufacturer’s instructions.

### Tumour xenograft mouse model

Animal ethics was granted by the Ethics Committee of Jinshan Hospital, Fudan University. Female 6- to 8-week-old BALB/c nude mice (Shanghai Super-B&K Laboratory Animal Corp. Ltd., Shanghai, China) were randomly divided into two groups: one group that received PNPO-shRNA-expressing cells (PNPO-shRNA) and the second group that received the parental SK-OV-3 NC cells (*n* = 14 per group). Animals were housed under standard 12/12 h light/dark cycle with free access to food and water. After culture, subconfluent PNPO-shRNA-expressing cells and control SK-OV-3 cells were resuspended, respectively, in DMEM without FBS. Approximately 5×10^6^ cells in 150 µl were injected into the right flank of each mouse. Body weight, tumour initiation and tumour progression were monitored every another day for 40 days (day of the first injection = day 0). Tumour volume was calculated using the formula *V* = *ab* ^2^/2^43^, where *V* is the volume, and *a* and *b* represent the tumour length and width, respectively. On day 40, animals were killed. Whole tumours were excised and photographed.

### Statistical analyses

All analyses were performed using SPSS Statistics 21.0 for Windows (SPSS, Chicago, IL, USA). For comparison of PNPO expression associated with the clinicopathological characteristics of patients with EOC, a Fisher’s exact test or *χ* ^2^ test was applied. For comparison between two groups in functional assays, vitamer detecting assays and treatment experiments, a Student *t*-test was used. Results are presented as the mean±standard error of the mean (SEM). A significant difference was considered at the value of *P* < 0.05.

## Electronic supplementary material

Supplementary Figure S1

Supplementary Figure S2

Supplementary Figure S3

Supplementary Figure S4

Supplementary Figure S5

Supplementary Figure S6

Supplementary Figure S7

Supplementary Figure S8

Supplementary Figure S9

Supplementary Table S1

Supplementary Table S2
