# Supplementary material for: Pyridoxine 5′-phosphate oxidase is a novel therapeutic target and regulated by the TGF-β signalling pathway in epithelial ovarian cancer
Source: Cell Death Dis. 2017 Dec 13;8(12):3214. doi: 10.1038/s41419-017-0050-3 (PMC5870590; doi:10.1038/s41419-017-0050-3)
Supplement: Supplementary file 1 — Supplementary Figure S1 [file 41419_2017_50_MOESM1_ESM.pdf]

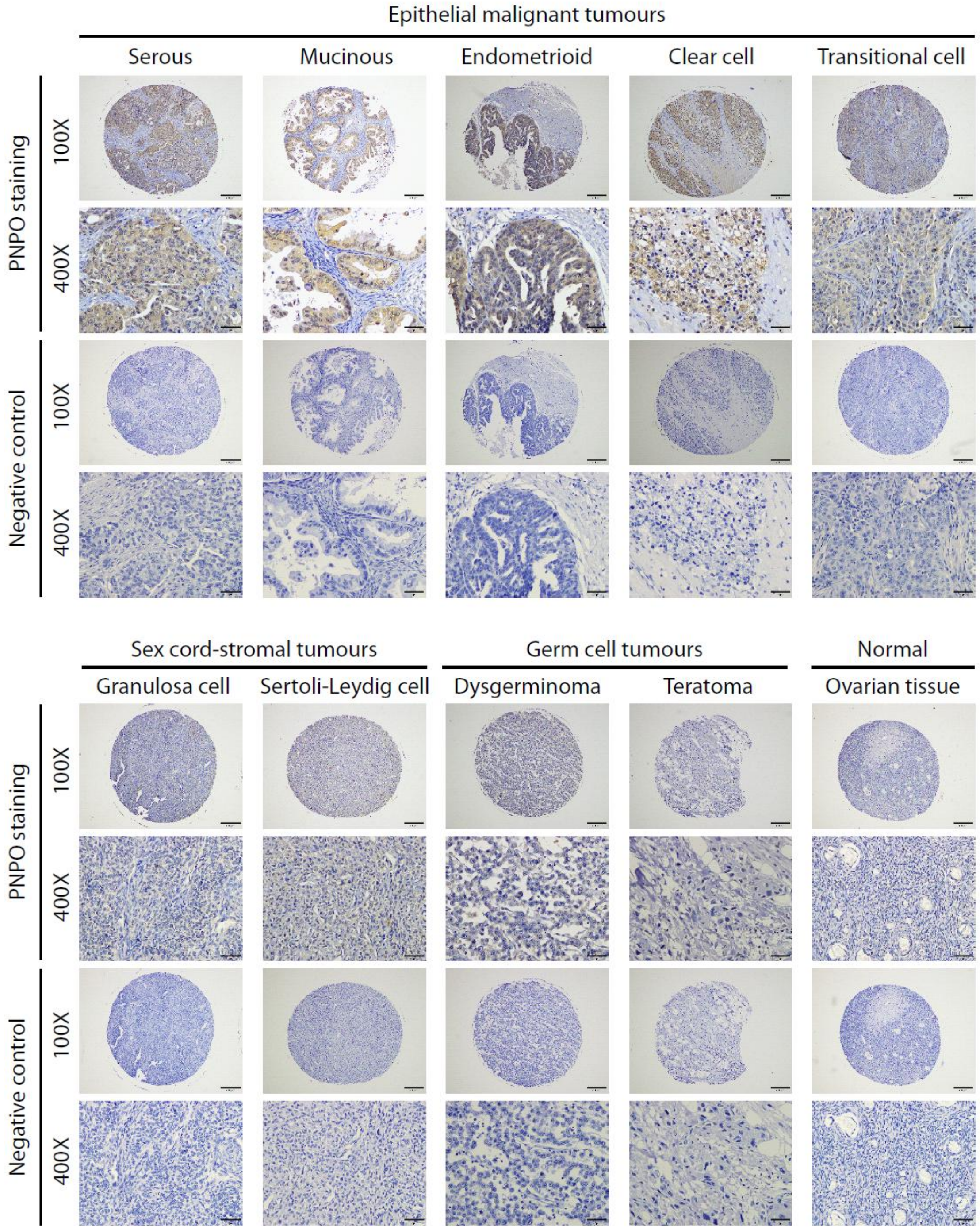

Supplementary Figure S1 PNPO expression of human ovarian tissues in a tissue microarray. Immunohistochemistry staining of PNPO protein was done using a specific antibody. Staining without a primary antibody was considered as the negative control. A brown color in a cell is considered as a positive staining. Representative images of PNPO expression in epithelial malignant tumours, sex cord-stromal tumours, germ cell tumours, and normal ovarian tissue are shown. Original magnification, ×100 and ×400.
