# Supplementary material for: Pyridoxine 5′-phosphate oxidase is a novel therapeutic target and regulated by the TGF-β signalling pathway in epithelial ovarian cancer
Source: Cell Death Dis. 2017 Dec 13;8(12):3214. doi: 10.1038/s41419-017-0050-3 (PMC5870590; doi:10.1038/s41419-017-0050-3)
Supplement: Supplementary file 2 — Supplementary Figure S2 [file 41419_2017_50_MOESM2_ESM.pdf]

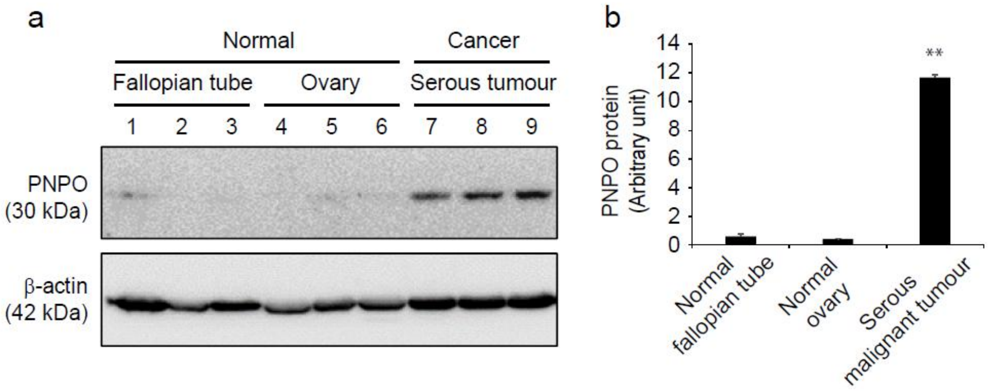

Supplementary Figure S2 PNPO protein expression. (a) PNPO protein was detected in the normal fallopian tube and ovarian tissues and epithelial serous malignant tumours by Western blot analysis using a specific antibody. (b) Semi-quantitative analysis of the relative optical density of protein bands in (a). Data are presented as mean ± SEM. **, P<0.001 in serous malignant tumours *vs.* normal controls; n=3 individuals.
