# Supplementary material for: Pyridoxine 5′-phosphate oxidase is a novel therapeutic target and regulated by the TGF-β signalling pathway in epithelial ovarian cancer
Source: Cell Death Dis. 2017 Dec 13;8(12):3214. doi: 10.1038/s41419-017-0050-3 (PMC5870590; doi:10.1038/s41419-017-0050-3)
Supplement: Supplementary file 3 — Supplementary Figure S3 [file 41419_2017_50_MOESM3_ESM.pdf]

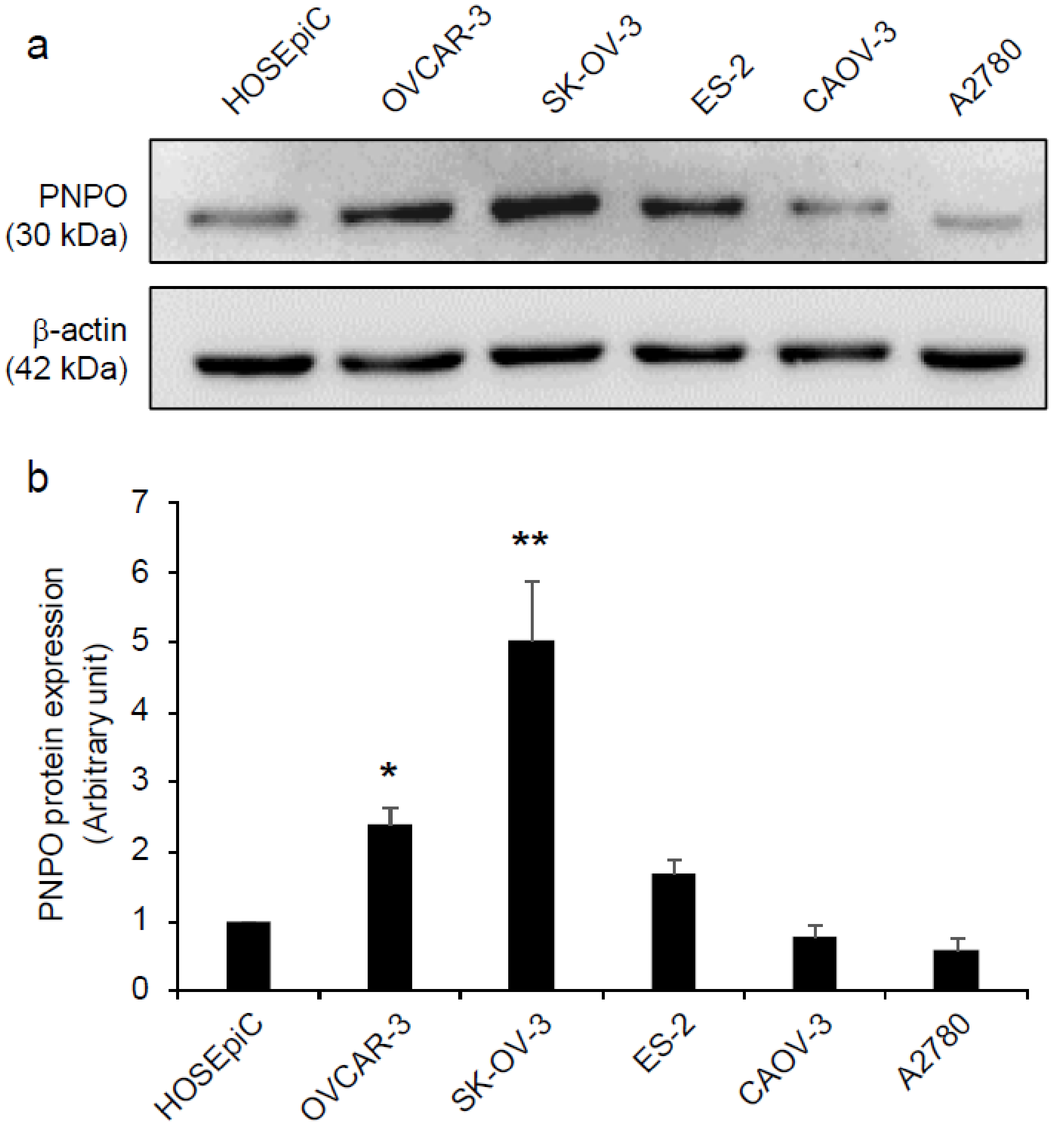

Supplementary Figure S3 PNPO protein expression in human ovarian surface epithelial cells. (a) PNPO protein was detected in HOSEpiC, OVCAR-3, SK-OV-3, ES-2, CAOV-3, and A2780 cells by Western blot analysis using a specific antibody. (b) Semi-quantitative analysis of the relative optical density of protein bands in (a). Data are presented as mean ± SEM. *, $P<0.05$ and **, $P<0.01$ in OVCAR-3 and SK-OV-3 *vs.* HOSEpiC controls; n=3 independent experiments.
