# Supplementary material for: Pyridoxine 5′-phosphate oxidase is a novel therapeutic target and regulated by the TGF-β signalling pathway in epithelial ovarian cancer
Source: Cell Death Dis. 2017 Dec 13;8(12):3214. doi: 10.1038/s41419-017-0050-3 (PMC5870590; doi:10.1038/s41419-017-0050-3)
Supplement: Supplementary file 4 — Supplementary Figure S4 [file 41419_2017_50_MOESM4_ESM.pdf]

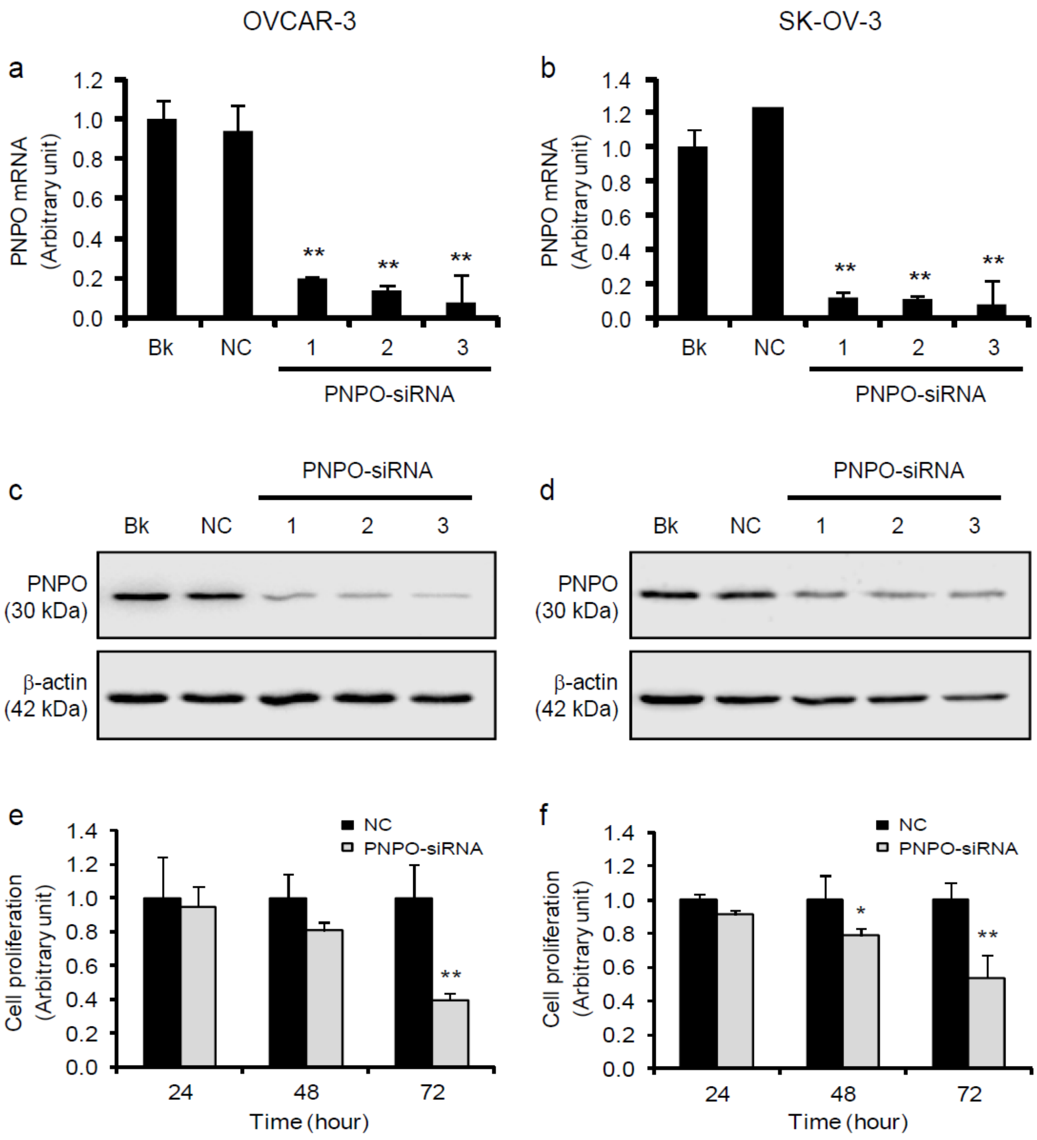

Supplementary Figure S4 Effect of PNPO on cell proliferation. (a, c, e) OVCAR-3 and (b, d, f) SK-OV-3 cells were transiently transfected with PNPO-siRNA #1, #2, and #3, respectively, or negative control-siRNA (NC). Untreated cells were used as a blank control (Bk). (a, b) PNPO mRNA expression was measured after 24 h post-transfection. (c, d) PNPO protein expression was detected after 48 hours post-transfection. (e, f) Cell proliferation was detected by the WST-1 assay after 24, 48, and 72 h post-transfection of PNPO-siRNA #2. Data are presented as mean ± SEM. *, $P<0.05$; **, $P<0.01$; n=3 independent experiments.
