# Supplementary material for: Pyridoxine 5′-phosphate oxidase is a novel therapeutic target and regulated by the TGF-β signalling pathway in epithelial ovarian cancer
Source: Cell Death Dis. 2017 Dec 13;8(12):3214. doi: 10.1038/s41419-017-0050-3 (PMC5870590; doi:10.1038/s41419-017-0050-3)
Supplement: Supplementary file 5 — Supplementary Figure S5 [file 41419_2017_50_MOESM5_ESM.pdf]

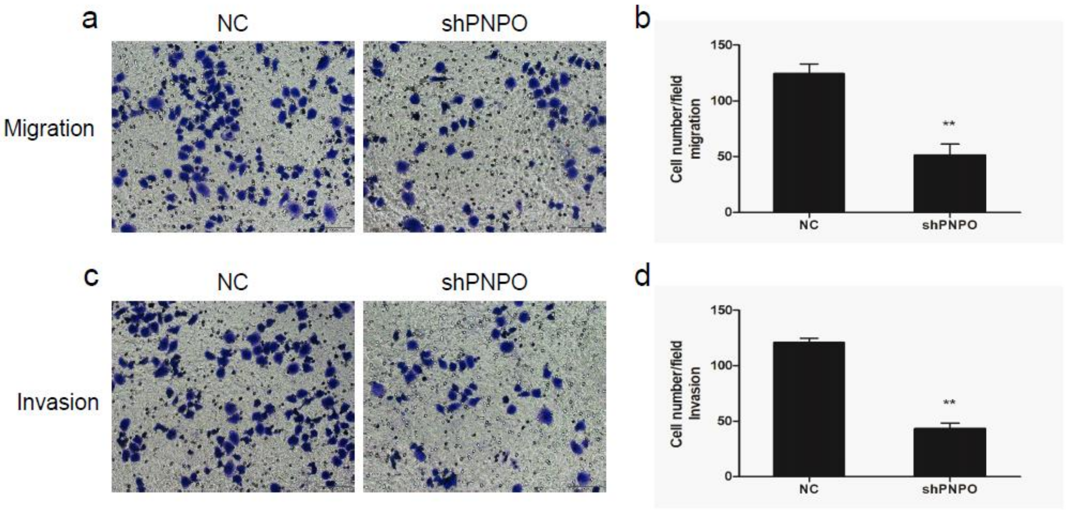

Supplementary Figure S5 Effect of PNPO-shRNA on cell migration and invasion. OVCAR-3 PNPO-shRNA-expressing cells (shPNPO) and negative control cells (NC) were used in experiments. (a) Transwell assay of cell migration. Photos were taken at 48 h after seeding. Original amplification, ×200. (b) Histogram shows the quantitative analysis of migrated cells of (a). (c) Cell invasion assay. Photos were taken at 48 h after seeding. Original amplification, ×200. (d) Histogram shows the quantitative analysis of invaded cells of (c). Data are presented as mean ± SEM. **, $P<0.01$; n=3 independent experiments.
