# Supplementary material for: Pyridoxine 5′-phosphate oxidase is a novel therapeutic target and regulated by the TGF-β signalling pathway in epithelial ovarian cancer
Source: Cell Death Dis. 2017 Dec 13;8(12):3214. doi: 10.1038/s41419-017-0050-3 (PMC5870590; doi:10.1038/s41419-017-0050-3)
Supplement: Supplementary file 6 — Supplementary Figure S6 [file 41419_2017_50_MOESM6_ESM.pdf]

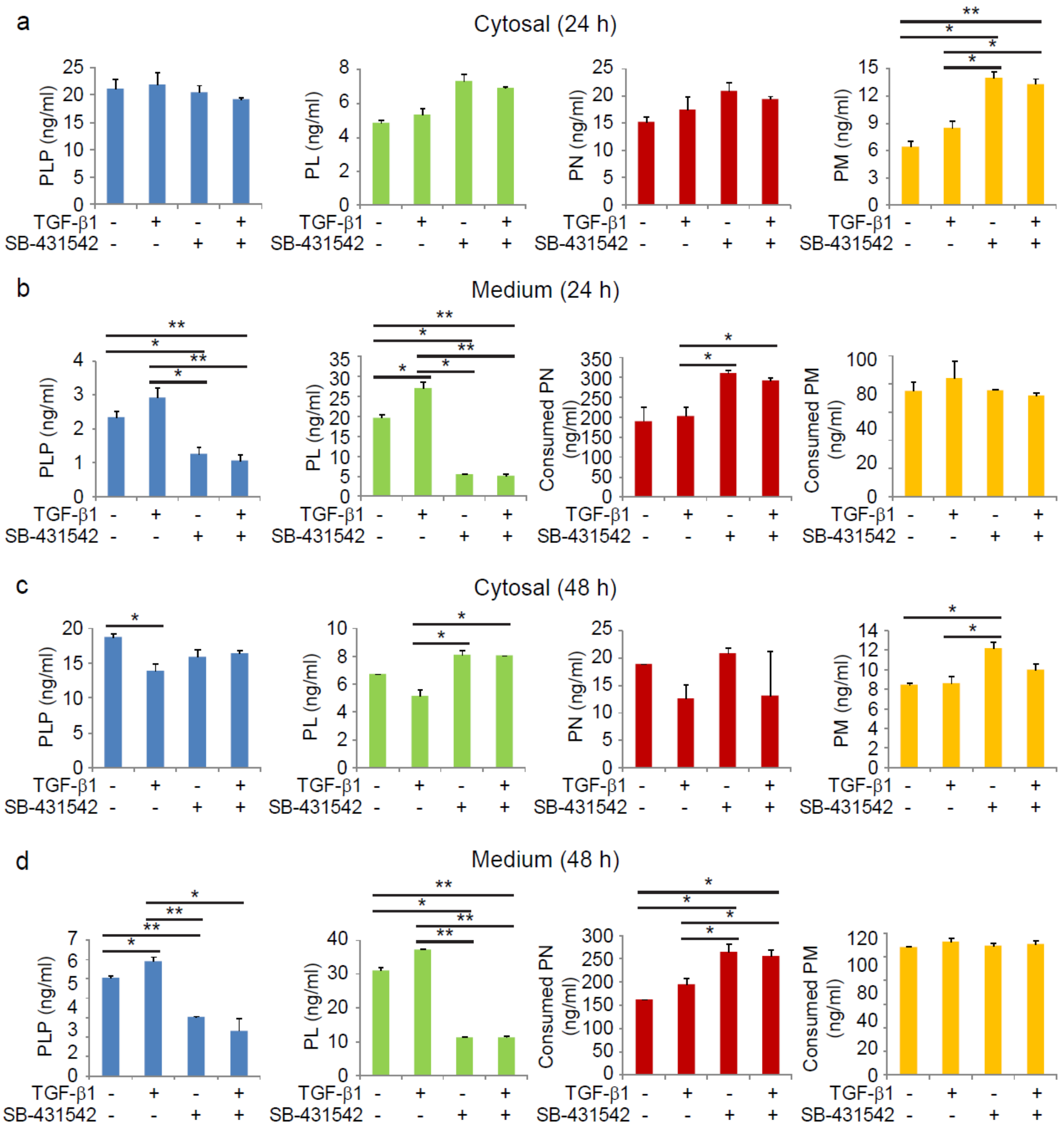

Supplementary Figure S6 Measurement of vitamin B6 vitamers in the cytosol and culture medium after TGF-β1 treatment. SK-OV-3 cells were cultured in the presence or absence of SB-431542 for 30 min, followed by the treatment of TGF-β1 (10 ng/ml) for 24 and 48 h. The concentration of vitamers PLP, PL, PN, and PM was measured. (a, b) Vitamer concentrations in the cytosol (a) and culture media (b) after TGF-β1 treatment for 24 h. (c, d) Vitamer concentrations in the cytosol (c) and culture media (d) after TGF-β1 treatment for 48 h. Data are presented as mean ± SEM (n=3). *, P<0.05; **, P<0.01; n=3 independent experiments.
