# Supplementary material for: Pyridoxine 5′-phosphate oxidase is a novel therapeutic target and regulated by the TGF-β signalling pathway in epithelial ovarian cancer
Source: Cell Death Dis. 2017 Dec 13;8(12):3214. doi: 10.1038/s41419-017-0050-3 (PMC5870590; doi:10.1038/s41419-017-0050-3)
Supplement: Supplementary file 7 — Supplementary Figure S7 [file 41419_2017_50_MOESM7_ESM.pdf]

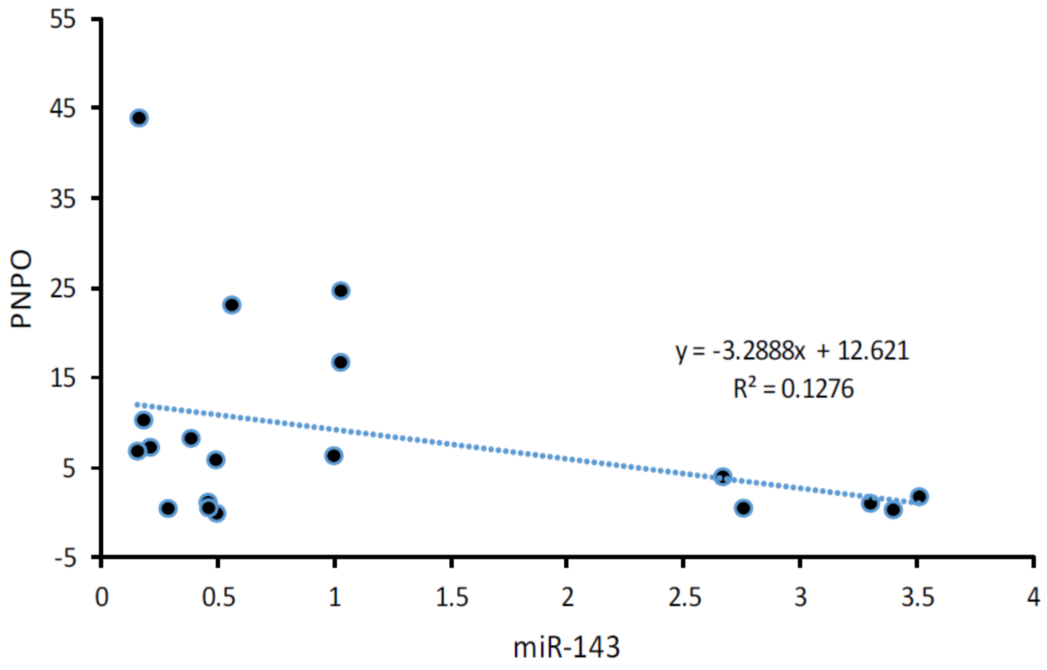

Supplementary Figure S7 Correlation between PNPO mRNA expression and miR-143 expression in patients with ovarian tumours. Total n=19 individuals (normal n=5, benign n=4, borderline n=3, malignant n=7).
