# Supplementary material for: Pyridoxine 5′-phosphate oxidase is a novel therapeutic target and regulated by the TGF-β signalling pathway in epithelial ovarian cancer
Source: Cell Death Dis. 2017 Dec 13;8(12):3214. doi: 10.1038/s41419-017-0050-3 (PMC5870590; doi:10.1038/s41419-017-0050-3)
Supplement: Supplementary file 8 — Supplementary Figure S8 [file 41419_2017_50_MOESM8_ESM.pdf]

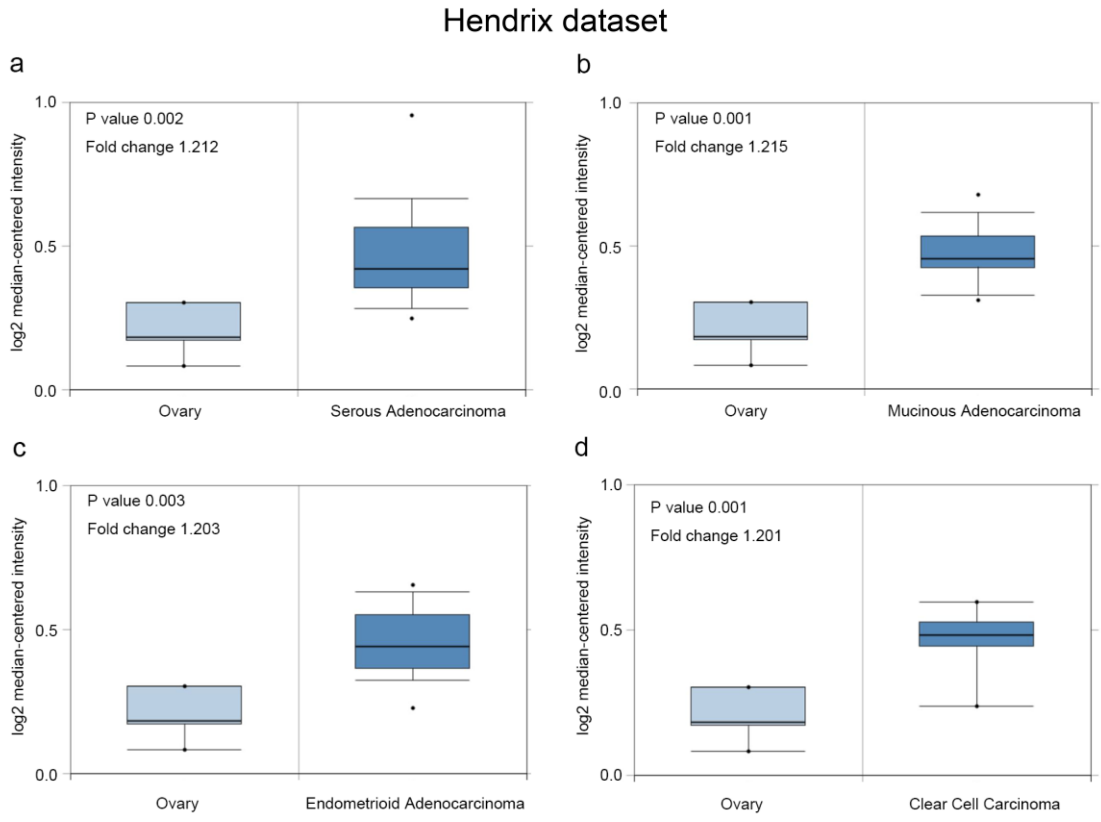

Supplementary Figure S8 PNPO mRNA expression in ovarian tissues. Data were obtained from the microarray datasets of Hendrix in Oncomine database (www.oncomine.org). High level of PNPO mRNA expression was found in (a) serous adenocarcinoma (n=41), (b) mucinous adenocarcinoma (13), (c) endometrioid adenocarcinoma (n=37), and (d) clear cell carcinoma (n=8) compared with normal ovary (n=4).
