# Supplementary material for: Pyridoxine 5′-phosphate oxidase is a novel therapeutic target and regulated by the TGF-β signalling pathway in epithelial ovarian cancer
Source: Cell Death Dis. 2017 Dec 13;8(12):3214. doi: 10.1038/s41419-017-0050-3 (PMC5870590; doi:10.1038/s41419-017-0050-3)
Supplement: Supplementary file 9 — Supplementary Figure S9 [file 41419_2017_50_MOESM9_ESM.pdf]

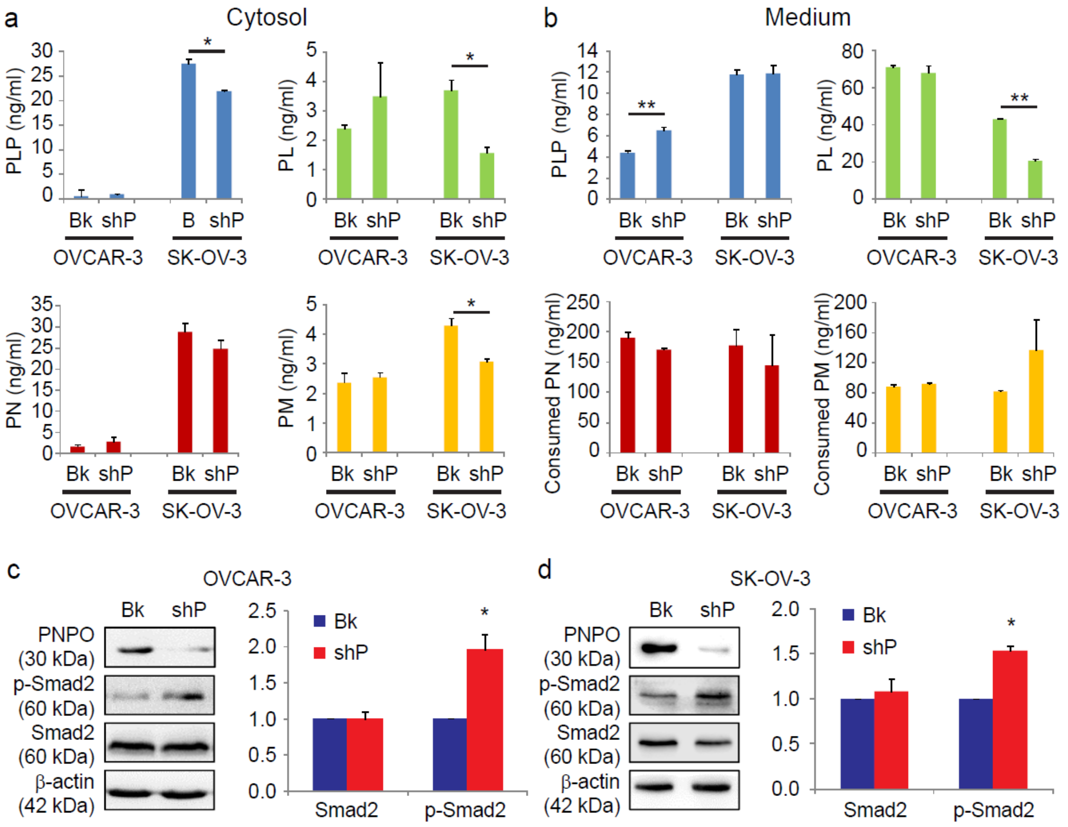

Supplementary Figure S9 Effect of PNPO-shRNA on vitamin B6 concentration and Smad2 phosphorylation. OVCAR-3 and SK-OV-3 cells were infected with PNPO-shRNA lentivirus to generate shPNPO expressing cells. The concentration of vitamers PLP, PL, PN, and PM in the cytosol (a) and culture media (b) were measured. Western blots of PNPO and Smad2 protein in OVCAR-3 (c) and SK-OV-3 (d) are shown, followed by the semi-quantitative analysis of the relative optical density of the protein bands. Data are presented as mean ± SEM. Bk, counterpart blank cells; shP, PNPO-shRNA expressing cells; *, $P<0.05$; **, $P<0.01$; n=3 independent experiments.
