# Supplementary material for: Pyridoxine 5′-phosphate oxidase is a novel therapeutic target and regulated by the TGF-β signalling pathway in epithelial ovarian cancer
Source: Cell Death Dis. 2017 Dec 13;8(12):3214. doi: 10.1038/s41419-017-0050-3 (PMC5870590; doi:10.1038/s41419-017-0050-3)
Supplement: Supplementary file 10 — Supplementary Table S1 [file 41419_2017_50_MOESM10_ESM.pdf]

Supplementary Table S1 Association of PNPO expression with the clinicopathological features of patients with tumours of the ovary

Clinicopathological features	n	PNPO expression		P-value
		Positive (%)	Negative (%)	
Age at diagnosis				0.015[a]
≤45	144	82 (56.94)	62 (43.06)	
>45	244	170 (69.67)	74 (30.05)	
Histological subtype				
Surface epithelial-stromal tumours				0.449[b]
Serous adenocarcinoma	227	167 (73.57)	60 (26.43)	
Mucinous adenocarcinoma	57	38 (66.67)	19 (33.33)	
Endometrioid adenocarcinoma	22	16 (72.73)	6 (27.27)	
Clear cell carcinoma	12	10 (83.33)	2 (16.67)	
Transitional cell carcinoma	5	5 (100.00)	0 (00.00)	
Sex cord-stromal tumors				1.000[b]
Granulosa-stromal cell tumours	24	7 (29.17)	17 (70.83)	
Sertoli-stroma cell tumours	2	0 (00.00)	2 (100.00)	
Germ cell tumours				0.669[b]
Primitive germ cell tumours	29	6 (20.69)	23 (79.31)	
Monodermal teratoma	10	3 (30.00)	7 (70.00)	
Grade				0.024[b]
1	89	55 (61.80)	34 (38.20)	
2	127	96 (75.59)	31 (24.41)	
3	81	64 (79.01)	17 (20.09)	
T stage				0.086[b]
T1	307	191 (62.21)	115 637.79)	
T2	43	33 (76.74)	10 (23.26)	
T3	38	28 (73.68)	10 (26.32)	
FIGO stage				0.196[b]
I	306	191 (62.42)	115 (37.58)	
II	35	27 (77.14)	8 (22.86)	
III	39	29 (74.36)	10 (25.64)	
IV	8	5 (64.96)	3 (35.05)	
LN metastasis				1.000[a]
No	27	18 (66.67)	9 (33.33)	
Yes	361	234 (64.82)	127 (35.18)	

The expression of PNPO was detected by immunohistochemistry. For comparisons of PNPO expression associated with age and LN metastasis, a Fisher's exact test was

applied ([a]). For multiple comparisons of PNPO expression associated with histological types, differentiation, and clinical stages, a χ^2 test was applied ([b]). n, number of cases; Positive, positive expression; Negative, negative expression; T, primary tumour site; FIGO, International Federation of Gynaecological Oncologists; LN, lymph node.
