# Supplementary material for: Pyridoxine 5′-phosphate oxidase is a novel therapeutic target and regulated by the TGF-β signalling pathway in epithelial ovarian cancer
Source: Cell Death Dis. 2017 Dec 13;8(12):3214. doi: 10.1038/s41419-017-0050-3 (PMC5870590; doi:10.1038/s41419-017-0050-3)
Supplement: Supplementary file 11 — Supplementary Table S2 [file 41419_2017_50_MOESM11_ESM.pdf]

Supplementary Table S2 Sequences of siRNA, shRNA, miRNA, and primer

Name	Sequence (5’ → 3’)	Target position
PNPO-siRNA		
Sense-1	GGAAAGAUGGCUUCCGCUUtt	nt 461-479
Antisense-1	AAGCGGAAGCCAUCUUUCCtt	
Sense-2	CCUUUGCUUCCCUUGUCUUtt	nt 527-545
Antisense-2	AAGACAAGGGAAGCAAAGGtt	
Sense-3	GGUGAUGGAGUUCUGGCAAtt	nt 795-813
Antisense-3	UUGCCAGAACUCCAUCACCtt	
NC-siRNA		
Sense	UUCUCCGAACGUGUCACGUTT	Scramble
Antisense	ACGUGACACGUUCGGAGAATT	
PNPO-shRNA		
Sense	gatccGACTGGCTCTATGAGAGACTTCAAG<br>AGAGTCTCTCATAGAGCCAGTCTTTTTTg	nt 910-928
Antisense	aattcAAAAAAGACTGGCTCTATGAGAGAC<br>TCTCTTGAAGTCTCTCATAGAGCCAGTCg	
miRNA		
miR-143 mimic	UGAGAUGAAGCACUGUAGCUC	nt 2198-2206
miR-Ctrl	UCACAACCUCCUAGAAAGAGUAGA	
anti-miR-143	GAGCUACAGUGCUUCAUCUCA	nt 2198-2206
anti-miR-Ctrl	UCUACUCUUUCUAGGAGGUUGUGA	
PNPO 3’-UTR construct primer		
Forward	gcgcccgcgatcgcgaattcGGGACCCACAGAATTA<br>CAGG	nt 2158-2177
Reverse	ctgctcgaactagtctcgagCCCAGGCTGCATTCATA<br>TC	nt 2618-2636
PNPO PCR primer		
Forward	TTGAGGAGACTCATCTGACC	nt 296-315
Reverse	GTTAGTGAAGAAGCGGAAGC	nt 470-489
β-actin PCR primer		
Forward	ACAATGTGGCCGAGGACTTT	nt 1412-1431
Reverse	GCACGAAGGCTCATCATTCA	nt 1654-1672
Pri-miR-143 primer		
Forward	GAAGCAAGAACTCTGGAGAAGC	nt 346-367
Reverse	GGACCTCTGTCTTCTGTTGG	nt 444-463
miR-143 primer		
Forward	CTGAGATGAAGCACTGTAGCTC	
Reverse	GTGCAGGGTCCGAGGT	
U6 primer		
Forward	CTCGCTTCGGCAGCACA	
Reverse	AACGCTTCACGAATTTGCGT	
miR-143 RT primer	GTCGTATCCAGTGCAGGGTCCGAGGTATT<br>CGCACTGGATACGACgagcta	

The position in PNPO mRNA sequence (GenBank Accession: NM_018129), β-actin mRNA sequence (GenBank Accession: NM_001101), and Pri-miR-143 (GenBank Accession: NR_105059) is shown. The low case indicates a linker. Underline indicates a site of restriction enzyme *EcoR* I or *Xho* I. NC, non-specific control; nt, nucleotide; PCR, polymerase chain reaction; shRNA, short hairpin RNA; siRNA, small interfering RNA.
